# Colon Cryptogenesis: Asymmetric Budding 

**DOI:** 10.1371/journal.pone.0078519

**Published:** 2013-10-21

**Authors:** Chin Wee Tan, Yumiko Hirokawa, Bruce S. Gardiner, David W. Smith, Antony W. Burgess

**Affiliations:** 1 The Walter and Eliza Hall Institute of Medical Research, Parkville, Victoria, Australia; 2 Department of Medical Biology, University of Melbourne, Parkville, Victoria, Australia; 3 Ludwig Institute for Cancer Research, Melbourne-Parkville Branch, Parkville, Victoria, Australia; 4 School of Computer Science and Software Engineering, University of Western Australia, Perth, Western Australia, Australia; 5 Department of Surgery, University of Melbourne, Parkville, Victoria, Australia; University College London, United Kingdom

## Abstract

The process of crypt formation and the roles of Wnt and cell-cell adhesion signaling in cryptogenesis are not well described; but are important to the understanding of both normal and cancer colon crypt biology. A quantitative 3D-microscopy and image analysis technique is used to study the frequency, morphology and molecular topography associated with crypt formation. Measurements along the colon reveal the details of crypt formation and some key underlying biochemical signals regulating normal colon biology. Our measurements revealed an asymmetrical crypt budding process, contrary to the previously reported symmetrical fission of crypts. 3D immunofluorescence analyses reveals heterogeneity in the subcellular distribution of E-cadherin and β-catenin in distinct crypt populations. This heterogeneity was also found in asymmetrical budding crypts. Singular crypt formation (i.e. no multiple new crypts forming from one parent crypt) were observed in crypts isolated from the normal colon mucosa, suggestive of a singular constraint mechanism to prevent aberrant crypt production. The technique presented improves our understanding of cryptogenesis and suggests that excess colon crypt formation occurs when Wnt signaling is perturbed (e.g. by truncation of adenomatous polyposis coli, APC protein) in most colon cancers.

## Introduction

The mucosal surfaces of the colon and rectum contain the openings to small invaginations known as crypts [1,2]. In the small intestine, it has been determined that the stems cells reside near the base of the crypts either at the ‘+4 position’ [3-5] between the paneth cells and the proliferative progenitor cells or juxtaposed to the paneth cells at the crypt base (these cells have been identified as crypt base columnar cells [6-11]). In the colon there are no identifiable Paneth cells, however based on the expression of the stem cell marker leucine-rich-repeat-containing G-protein-coupled receptor 5 (LGR5) [12], the colon stem cells also appear to reside at the crypt base[13]. 

Most discussions of intestinal homeostasis overlook the fact that there are two processes responsible for maintenance of the intestinal mucosa: continuous cell production within a crypt [14] and the production of new crypts [15]. The production of new crypts from stem cells is generally believed to be brought about by a symmetrical process known as fission [15-18] (“the longitudinal splitting of intestinal crypts starting from the bottom near the muscularis mucosa and proceeding upwards toward the villus with the subsequent production of two crypts” [15]). However, neither the morphology of crypt fission nor the processes controlling crypt production is well described. In mice, the frequency of fission decreases with age [15,19]. Our results suggest new crypts are actually produced by budding from cells near the base of the crypt. The concept of different types of crypt stem cells has been debated for many years [5,7,12,20], however, these discussions have focused on self-renewal and cell production within a crypt. It have been described in intestinal crypts that there are at least two classes of stem cells, one quiescent and the other actively dividing [5,12]. However, their correlations with functional outcomes are still unclear (i.e. crypt production or crypt maintenance). Techniques to allow characterization of the markers and corresponding functions of intestinal stems cells under homeostatic conditions, wound repair or oncogenic transformation are essential. 

The Wnt signaling pathway is involved in cell proliferation and regulation of homeostasis in the intestine [21,22]. Wong and colleagues noted, in their histology studies on human colorectal cancer, that crypt fission and cell proliferation appear to play critical roles in the growth of colorectal adenomas and hyperplastic polyps [23]. A recent study on infant rat intestinal mucosa reported that inhibition of Wnt signaling by Dickkopf-1 reduces crypt fission [24]. *In vitro*, cell production in neonatal mouse colon organoids was also reduced by Dickkopf-1 [25]. Hirata and colleagues have also implicated canonical Wnt signaling in crypt formation and cell proliferation in mice [21]. Although it is accepted that the coordination of cell production and differentiation is accomplished via a combination of cellular signaling pathways [22,26], other processes (e.g. mechanical forces between neighboring cells, cell-matrix interactions, rate of cell migration, mucous production and cell death) also have significant roles in maintaining the crypt structure. The subcellular distribution of Wnt responsive and/or cell-cell adhesion proteins in intact colonic crypts will improve our understanding of these pathways and their roles in the production and maintenance of crypts. Previous reports on β-catenin and/or E-cadherin expression in the colonic crypt, using immunohistology, have indicated a β-catenin expression gradient along the colonic crypt axis, with the highest expression at the crypt bottom [21] particularly for cytoplasmic and nuclear compartments [27]. E-cadherin expression has been reported to be a uniform, membrane localized protein in normal colonic mucosa, with reduced expression in adenomatous polyps [28]. 

**Figure 1:**
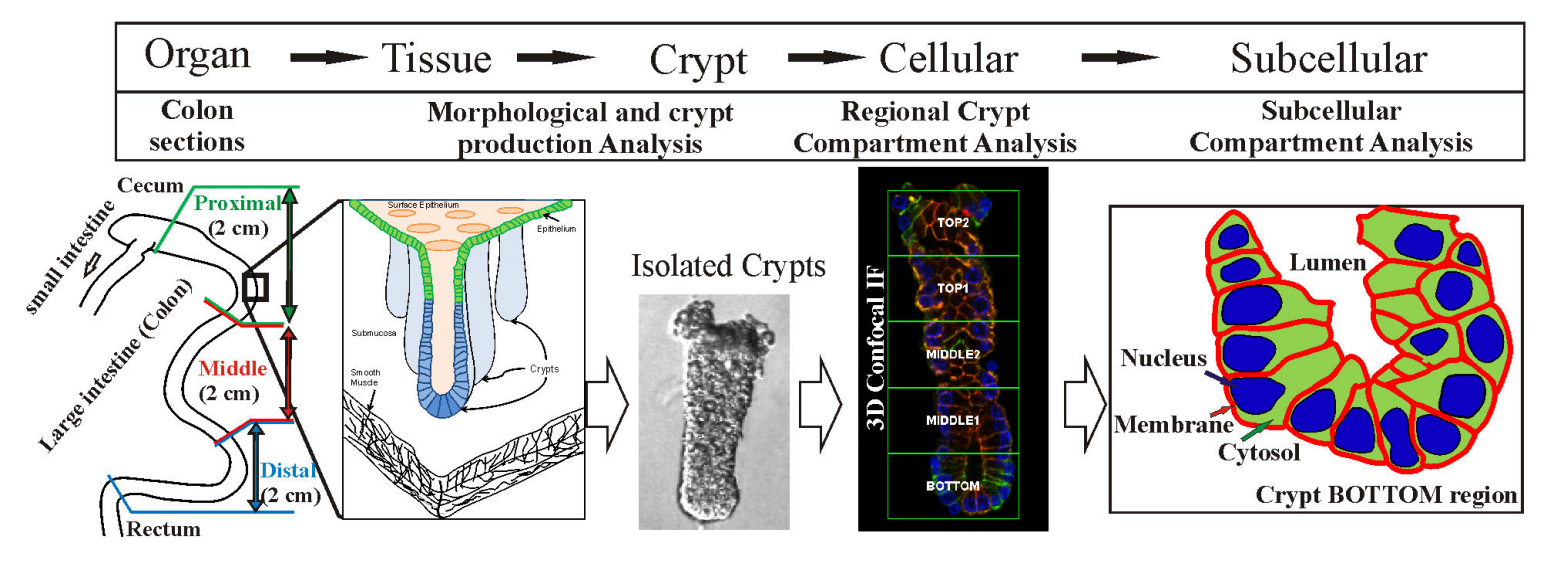
Schematic of the scale lengths and analyses used for analyzing crypt production in the colon. The mouse colon is considered in 3 regions between the caecum and the anus: Proximal, middle and distal. The epithelial surface is separated from the submucosa to produce isolated crypts. The morphology and cellular distribution along the crypt lumen axis are analyzed and the nuclear, cytoplasmic and membrane distribution of E-cadherin and β-catenin were determined. An understanding of the differences between regions of the colon, the morphology of the isolated crypts, the cell packing of the crypts and the subcellular compartments enables the structure and functional relationships of the underlying crypt formation and cell production to be determined.

In this study, a crypt isolation protocol (modified from Whitehead et al 1999 [29]) and a 3D-quantitative confocal imaging technique [30] were combined to interrogate crypt production and the subcellular distribution of E-cadherin and β-catenin across different length scales and compartments of the colon (see Figure 1). Using this approach, we investigated the morphology of the crypts isolated from different regions of mouse colon for different ages of mice. This technique also allowed a detailed analysis of crypt “fission” (budding) frequency and morphology. Our analysis indicated that the majority of the crypt production events are asymmetrical and result from budding. This technique enabled the frequency of crypt budding to be measured accurately. 

In order to understand key aspects of the underlying signaling biochemistry, we also used quantitative 3D confocal microscopy [30] to analyze the regional and subcellular distributions of E-cadherin and β-catenin in whole mount colonic crypts. The subcellular distribution of β-catenin and E-cadherin in cells along the length of the colonic crypt show different patterns. There are distinct clusters of cells at the base of the colonic crypt: in some clusters, the levels of E-cadherin are significantly reduced; in other clusters, the level of membrane E-cadherin and β-catenin are higher. Nuclear β-catenin levels are highest at the base of the crypt, while membrane levels of β-catenin are lowest in the middle regions of the crypt. In the small intestine, the Paneth cells have an accumulation of β-catenin in the nucleus [31], when compared to the other cells analyzed. Our results provide the first quantitative measurements of compartment β-catenin and E-cadherin protein levels along the length of colonic crypts. 

## Results

### Morphology of crypts differs along the length of the colon

**Figure 2:**
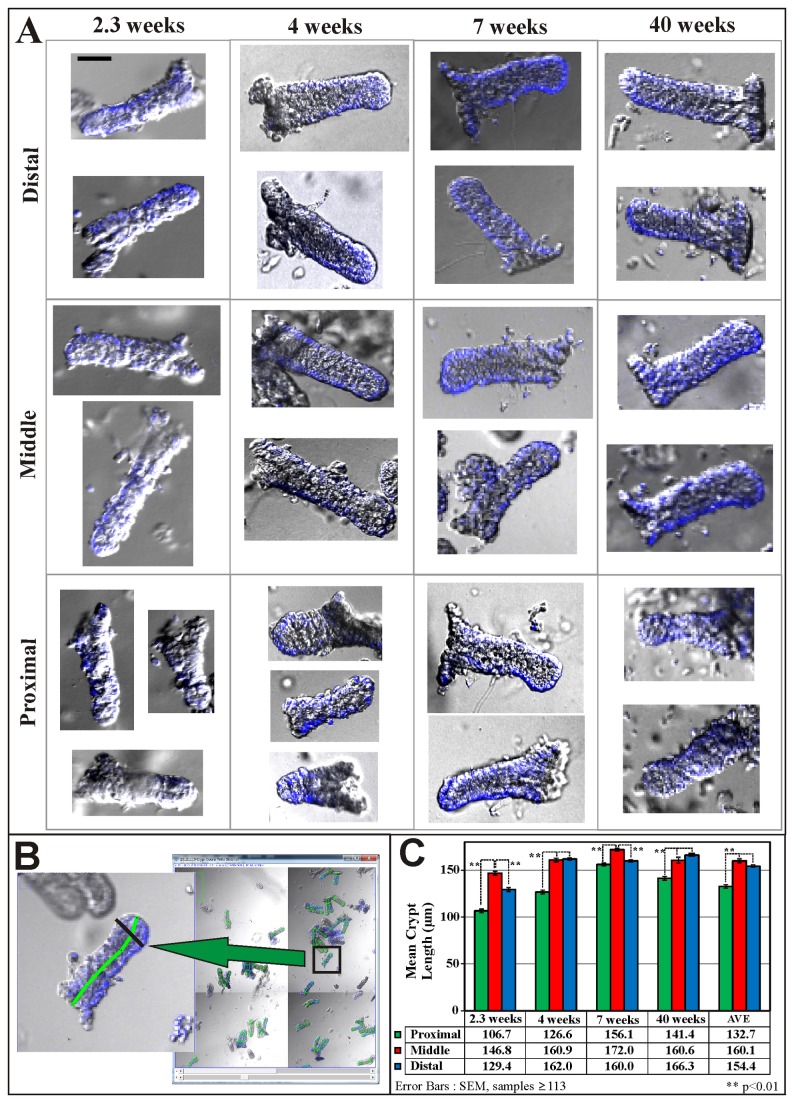
Confocal images of isolated proximal, middle and distal crypts from mouse colons of different ages. (A) Selected 2D confocal images of isolated colonic crypts from the different regions of the colon (proximal, middle and distal) obtained from mice of varying ages. The size and shape of crypts differ with the age of the mice and regions of the colon. Crypts isolated from very young mice (< 2 weeks) are fragile and easily broken. Images are of the same magnification with scale bar of 50µm. (B) Screen capture of a Fiji/ImageJ window with a typical image stack showing how the crypts are labeled, counted and measured. In this instance, a zoomed-in image of the crypt being measured is shown (middle panel) with a green segmented line marking the length of the crypt and a black segmented line marking the basal width of the crypt (drawn at about 20% of the crypt length from the crypt base). (C) Length measurements are made (in ImageJ as described in B) and tabulated for crypts isolated from mice aged 2.3, 4, 7 and 40 weeks. The proximal crypts are significantly shorter than the crypts from the middle or distal regions of the colon (p<0.01). Note: Images are 2D sections of image stacks consisting of a phase contrast image and DAPI staining in blue.

Using 3D confocal microscopy, we investigated the morphology of crypts isolated from different regions of the colon (Figure 1, colon sections). Proximal, middle and distal colonic crypts from mice of different ages (16 days up till adult 40 weeks) were isolated and analyzed (Figure 2). The lengths (Figure 2B) of more than two thousand crypts were measured. The images depict the morphology and structurally integrity of the crypts using our protocol. The crypts isolated from young mice (

< 2 weeks, before and during weaning) are fragile and break easily, crypts from older mice (> 2 weeks, after weaning) are structurally more robust and maintain their shape during the isolation process. The fragility of crypts from the young mice limited the application of the morphological (length and production rate) analysis to mice greater than 2 weeks of age. The best age for isolation and analysis of crypt production is between 4 to 7 weeks old: these crypts are structurally intact and there is a high rate of crypt formation. Crypts from the proximal colon are significantly shorter (133±28µm) than the crypts from either the middle (160±32µm) or distal (154±21µm) colon for mice between 2.6 and 40 weeks old (Figure 2C). 

### Asymmetrical crypt budding process

**Figure 3:**
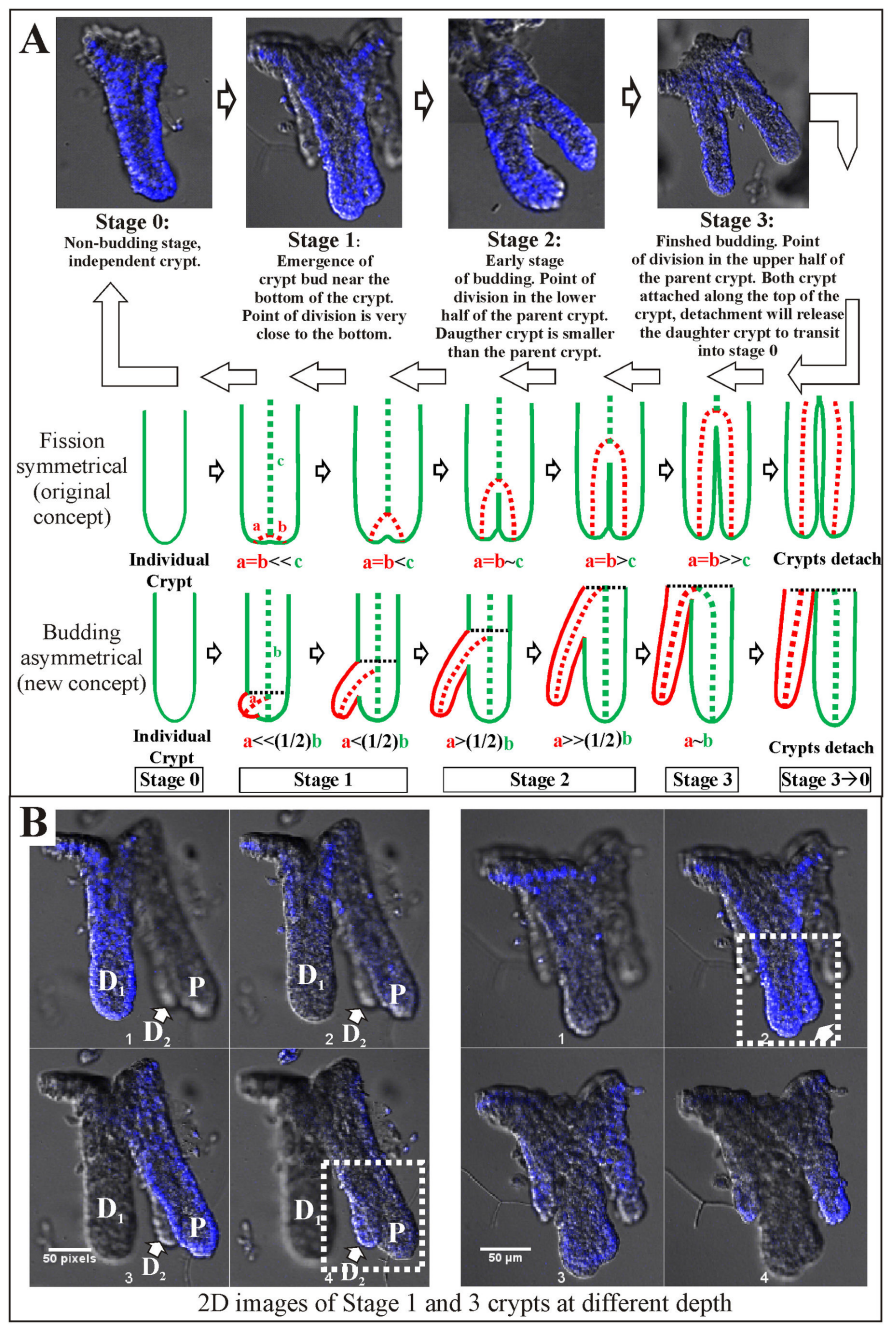
Stages of colon crypt development and budding rates for mice of different ages. (A) 3D confocal image of colonic crypts at the four stages of crypt development (defined as stages 0 to 3) as proposed in this study. An asymmetrical crypt production concept is proposed (budding), contrary to the symmetrical concept (fission). Schematic representations of the two concepts: red representing the development of a new bud; green sections depict the continuation of the original crypt; black dotted line represents the developing front of the crypt(s). Stages are defined based on the position of the new crypt base and the point of division of the crypts as described below the representative confocal image of each stage. Stage 0 is the only non-budding phase; Stage 1 represents the onset of crypt bud formation; Stage 2, in the midst of budding and Stage 3, late completion stage of budding. Stage 3 crypts transits into independent stage 0 crypts upon detachment from each other. (B) 2D images from image stacks showing the advantage provided by confocal 3D imaging of isolated crypts in capturing previously undetected buds of colonic crypts that are in stage 1 (white arrows). Onset of budding is difficult to detect using micro-dissection [17,32,33] or serial tissue sectioning [34]. Asymmetrical budding is also shown to occur in stage 1. P: Parental Crypt, D1: Daughter crypt 1, D2: Daughter crypt 2.

On the basis of the 3D confocal images of the isolated colonic crypts, four stages (0-3) of colonic crypt development were defined. In this paper, the original crypt is termed “parent” and the new crypt “daughter” (typically smaller in basal width). Figure 3A illustrates crypt development from a non-producing crypt (Stage 0), to the start of crypt production with the appearance of a small protrusion (bud) near the bottom of the crypt (Stage 1), to the middle of the crypt formation process (Stage 2) where the daughter crypt protrudes from the parent crypt, until the new crypt reaches the top half of the parent crypt (finished crypt formation, Stage 3). Our observations indicate that crypt formation is asymmetrical in stage 1 (Figure 3A). It has been generally accepted that new intestinal crypts are produced by symmetrical fission [17,18], however, we propose an asymmetrical crypt formation process with the new crypt starting near the base of the crypt (Figure 3A, schematic). Typically, stage 2 is the “fission” phase identified in previous studies [17,32-34] that used micro-dissection or serial tissue sectioning methods. Small buds at the start of the process (Stage 1) are difficult to detect using those methods and therefore were generally not measured [17,32-34]. Using the confocal imaging technique (15-20µm thick sections) on isolated crypts allows the small buds (Stage 1) to be detected and counted (Figure 3B, white dotted boxes). See Text S2 and Figure S5 for discussions and validations of the crypt isolation and imaging technique used in this study as well as a comparison of the different analysis techniques. 

Our observations (Figure 3) show an asymmetric new crypt protrusion emerging (Stage 1) near the bottom of the crypt (no new crypt protrusions emerge from the top of a parent crypt). The protrusion gradually grows along the length of the parent crypt (Stage 1-2). The developing front (Figure 3A, black dotted line) of the crypt progresses upwards from near the bottom of the parent crypt to the top of the crypt presumably with the maturing cells of the parental crypt. By Stage 3, the developing front has reached the top half of the parent crypt with the daughter crypt still attached. The daughter (D_1_) and parental (P) crypts remain attached while the daughter crypt matures. We have observed that in some cases, the parent crypt can also start a new crypt protrusion (D_2_) while the daughter crypt (D_1_) is still attached in Stage 3 (i.e. Stage 1 and Stage 3 together, see Figure 3B) where the initial crypt formation has completed. However, no multiple immature daughters (i.e. stage 1 or 2) have been observed in crypts isolated from the normal colon mucosa. The completion from stage 0 to 3 may present a necessary constraint during normal crypt formation, preventing aberrant crypt production.

**Figure 4:**
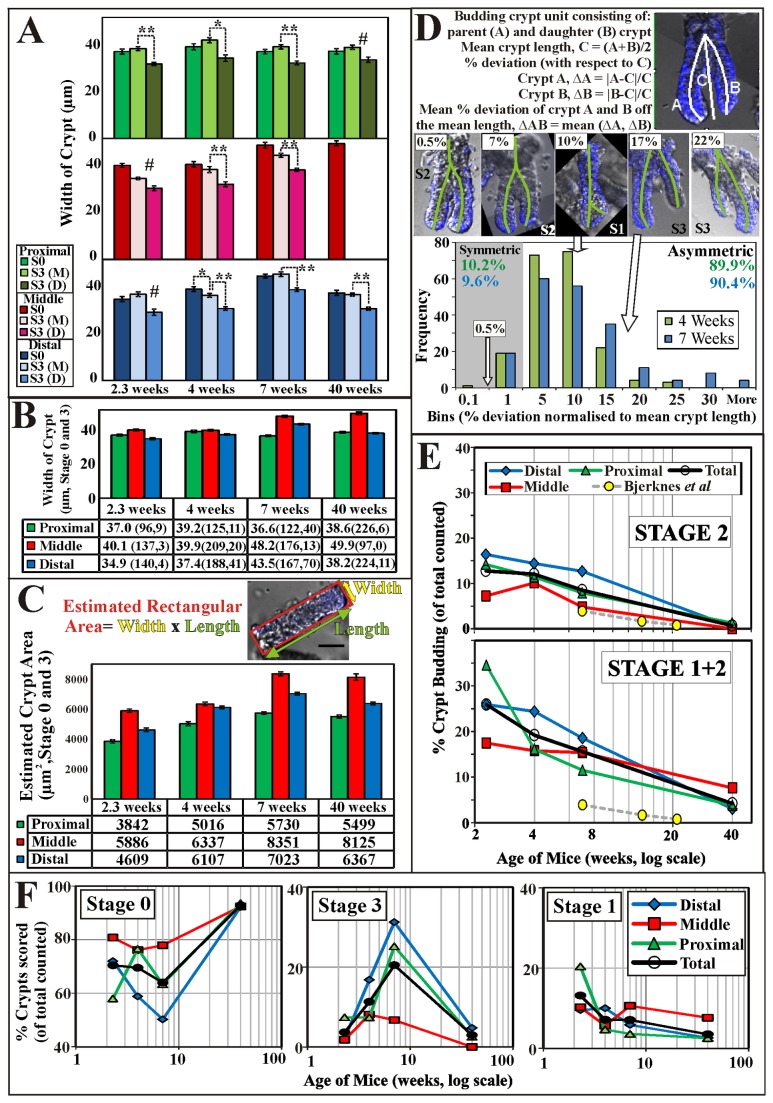
Measurements and analysis of colon crypt production. (A) Mature crypts (Stage 0 + 3) are similar in length while the daughter crypt (Stage 3) is significantly shorter than the mature crypt in different colon regions and animal age (* p<0.05, **p<0.01, #daughter crypt count < 10, S0-Stage 0, S3-Stage 3, M- mature crypts, D- daughter crypts). (B) The average basal width of isolated crypts (Stage 0+3) for the different regions of the colon showing crypts from the middle regions are wider than the other regions. (C) Using an estimated rectangular area as a measure of crypts size, the average crypt widths and lengths (stage 0+3) from the proximal, middle and distal regions for mice aged 2.3, 4, 7 and 40 weeks are multiplied. The crypts from the middle regions are the largest with the proximal crypts the smallest for each age group. (D) Derivations for asymmetry index (mean % deviation, AI) describing the degree of crypts budding asymmetrically is shown. 2D DAPI-labeled images of crypts at different level of asymmetry shown with corresponding AI labeled above the image. Using 1% AI as the cut-off to approximate symmetry during budding, a histogram of the AI frequency of crypts (4 and 7 weeks of age) indicates ~ 90% (89.9% and 90.4%) of the crypts has AI greater than 1%. (E) “Stage 2” and “Stage 1+2” crypt budding rates, showing a significant decrease with mice age. The “Stage 2” analysis was used as a comparison with previously reported numbers of crypt budding using micro-dissection [17,32,33] or serial sectioning [34] (where Stage 1 buds are difficult to detect). Crypt budding rates for the proximal, middle and distal regions of the colon are compared to that reported by Bjerknes et al [35]. (F) Crypts scored in Stages 0, 1 and 3 of the study are tabulated.

It is noted that the daughter crypt is smaller than the parent crypt during stage 2/3, and increases in size as it matures. Comparing the basal widths of ‘mature’ crypts (from stage 0 and 3) to smaller ‘daughter’ crypts from stage 3 (Figure 4A) shows that daughter crypts have significantly shorter basal widths than the mature crypts. The average basal width of mature crypts (i.e. wider S0 and S3 wider crypts) are very similar (not significantly different), indicating a consistent basal width upon maturity (Figure 4A). The crypts from the middle regions have the largest basal width (Figure 4B). In fact, by approximating the size of crypts using a rectangular area (length x width), the crypts in the middle regions of the colon appear to be the largest and proximal crypts the smallest (Figure 4C).

To quantitate the asymmetry of crypt formation, a simple asymmetry index (AI, in the form of the percentage deviation of the lengths of the individual branches of the budding crypts) is identified for stage 1 and 2 crypts for mice between the age of 4 and 7 weeks (Figure 4D). The majority (about 90%) of the colon crypts buds are produced by an asymmetric process. We termed this asymmetrical crypt production “crypt budding” to distinguish it from the symmetrical crypt fission process in the literature [17,18]. 

### Colonic Crypt budding rates decrease with age

Crypt production data from previous studies (using micro-dissection [17,32,33] or serial tissue sectioning [34] techniques) varies substantially with the different methods of isolation, measurement, regions of colon and mouse strain. For example, Bjerknes et al [35] used EDTA perfusion to isolate colon crypts to estimate branching (fission) crypts fraction in normal (C57BL/6J) and APC^MIN^ mice. They reported fission rates of 4% for 50 days (~7 weeks) old mice, 1.7% for 100 days (~14 weeks) old mice and 0.8% for 150 days (~21 weeks) old mice. As mentioned previously, stage 1 budding crypts are difficult to detect using these methods without confocal sectioning. Crypt production frequencies for the stage 2 crypt buds scored in this study are compared to the data reported by Bjerknes et al [35] for older mice (Figure 4E, top panel). From the comparison presented in Figure 4E, it is clear that in mice there is decreasing crypt budding frequency with increasing age. This is similar to those reported in the Bjerknes et al study, however, the numbers of crypt budding events reported by Bjerknes et al are lower than those measured in this study. When stage 1 crypt budding frequency (Figure 4E, Stage 1+2 bottom panel) is included, our budding rates are significantly higher than previously reported. However, in agreement with Bjerknes et al, it is clear from the measurements made in this study that the crypt production frequency decreases with the age of the mouse (Figure 4E). 

For mature crypts in stage 0, the higher frequencies of stage 0 (proportion of total crypts scored) occur in young mice (2 to 3 weeks) and adult mice (40 weeks, Figure 4F). During development (4 to 7 weeks), the frequencies of stage 0 crypts decrease. The frequencies of Stage 3 crypts is the reverse of that observed for Stage 0 crypts, with higher frequencies occurring during development (4 to 7) and reduced frequencies in young and adult mice (Figure 4F). The proportions of the budding crypts (stages 1 and 2) to mature crypts (stages 0 and 3) for different ages of mice are tabulated in Table 1. Budding rates (Stage 1 and 2) decrease with animal age while non-budding rates (Stage 0 and 3) increase with animal age (Table 2).

**Table 1:** Frequencies of colonic crypt in different crypt formation stages for different age of mice.

		**% budding of Total Crypts Counted**															
		Stage 1				Stage 2				Stage 3				Stage 0			
		Budding								Non-Budding							
**Age of Mice (Weeks)**	Total Crypt Counted	D	M	P	T	D	M	P	T	D	M	P	T	D	M	P	T
**2.3**	517	9.5	10.2	20.4	13.2	16.4	7.2	14.2	12.8	2.1	1.8	7.4	3.7	72.0	80.7	58.0	70.4
**4.0**	646	10.0	5.7	4.7	7.1	14.4	10.1	11.4	12.1	16.8	8.1	7.4	11.3	58.8	76.1	76.5	69.5
**7.0**	552	5.9	10.6	3.6	7.1	12.7	4.8	7.9	8.5	31.2	6.7	25.2	20.5	50.2	77.9	63.3	63.9
**40.0**	571	2.6	7.6	2.6	3.5	0.4	0	1.3	0.7	4.8	0	2.6	3.0	92.2	92.4	93.6	92.8

* D: Distal ; M: Middle ; P: Proximal ; T: Total

**Table 2:** Budding and non-budding frequencies of colonic crypt for different age of mice (% budding of Total Crypts Counted).

**Age of Mice**	**Budding (Stages 1+2)**				**Non-Budding (Stages 0+3)**			
**Weeks**	D	M	P	T	D	M	P	T
**2.3**	25.9	17.5	34.6	25.9	74.1	82.5	65.4	74.1
**4.0**	24.4	15.8	16.1	19.2	75.6	84.2	83.9	80.8
**7.0**	18.5	15.4	11.5	15.6	81.5	84.6	88.5	84.4
**40.0**	3.0	7.6	3.8	4.2	97.0	92.4	96.2	95.8

* D: Distal ; M: Middle ; P: Proximal ; T: Total

### Spatial sub-cellular distribution for β-catenin and E-cadherin

**Figure 5:**
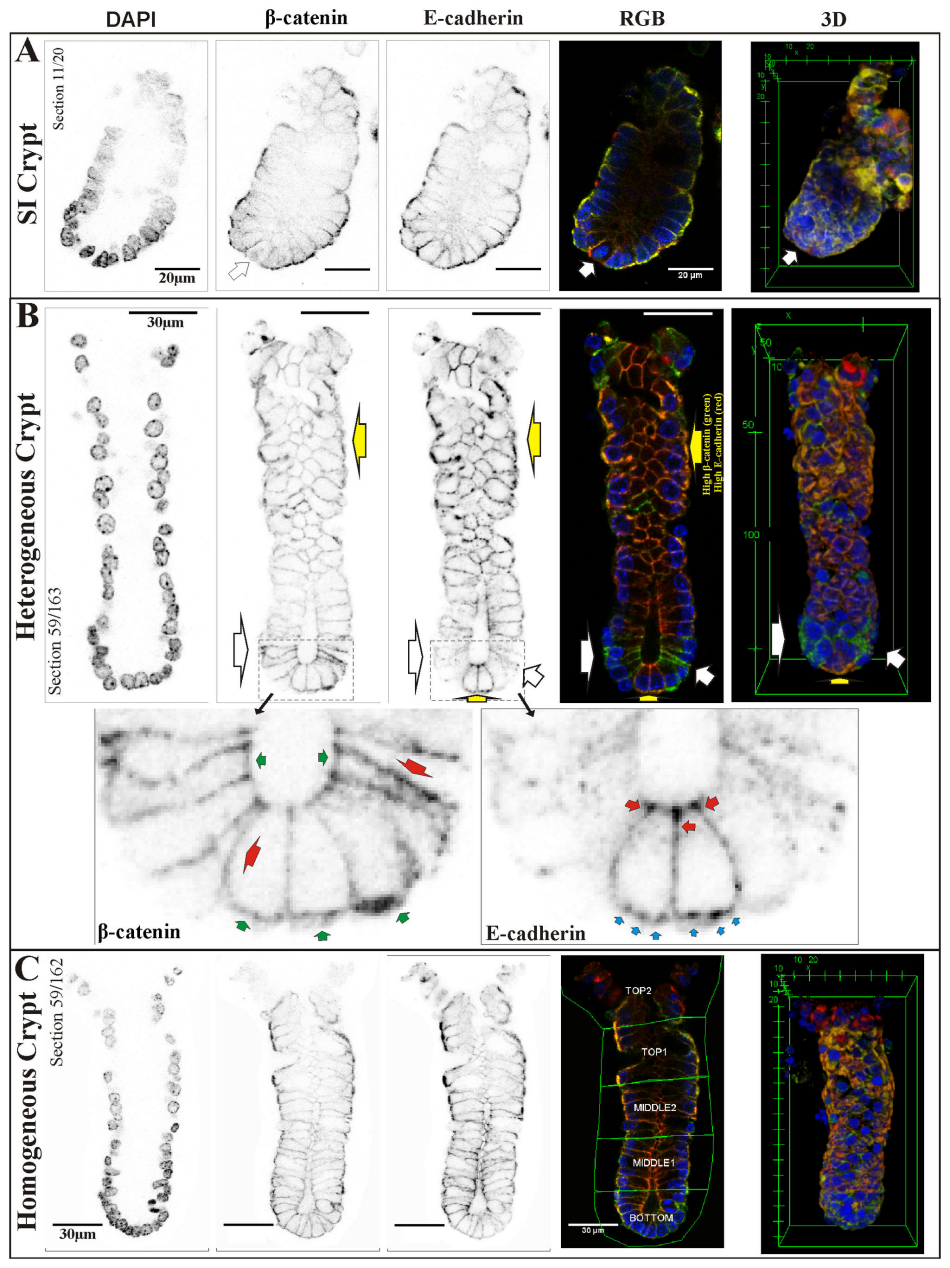
β-catenin and E-cadherin expression in small intestine and colonic crypts. Immunofluorescent 2D images extracted from 3D image stacks of one small intestinal crypt (A) and two isolated colonic crypts (B-C) with the DAPI, β-catenin, E-cadherin, overlayed RGB images and 3D view shown (see supporting information for movies and data sets). (A) Validation of the immuno-staining was conducted by applying the same isolation and analysis protocol on small intestinal crypts with panel A showing the corresponding 2D images of DAPI, β-catenin, E-cadherin and overlaid RGB images from one 3D image stack of a small intestinal crypt. The high levels of β-catenin in the nucleus of a Paneth cell (white arrow) are typical of the small intestinal crypts [31]. (B) For 60% of the colon crypts (heterogeneous population, HE crypts) scored, β-catenin expression is mainly present at the membrane along the whole length of the crypt. E-cadherin exhibit: “bands” or “rings” of E-cadherin low expressing cells (white arrows) at the lower half of the crypt leading to distinct clusters of high E-cadherin expressing cells at the bottom surrounded by the low expression clusters (yellow arrow). Zoomed images of β-catenin and E-cadherin at the crypt bottom; β-catenin localizes to the lateral (red arrows) membrane with some apical or basal (green arrows) localization observed. E-cadherin localizes to lateral-apical (red arrows) and basal membrane (blue arrows). (C) For the remaining 40% of the colon crypts (homogenous population, HO crypts) scored, β-catenin and E-cadherin expression appears to be present homogenously at the membrane along the whole length of the crypt. Note: Crypt compartments shown in C, RGB panels (5 evenly divided regions of interest); Inverted black color used in place of individual color for clarity. Images digitally scaled for display purposes using Fiji/ImageJ [68].

To relate underlying biochemistry to crypt morphology and function, spatial expression levels and localization of β-catenin and E-cadherin in the colonic crypts were investigated using high resolution 3D confocal imaging and analysis. Confocal imaging experiments with 32 fluorescent stained colonic crypts were analyzed. Small intestinal crypts were also stained and analyzed as a control. High levels of β-catenin are present in the nucleus of some of the larger cells at the base of the small intestine crypts (see Figure 5A). We presume these to be Paneth cells, as high levels of nuclear β-catenin have been reported for Paneth cells [31]. These cells have low levels of membrane β-catenin, so they appear red (i.e. E-cadherin only).

In the colonic crypts (Figure 5B-C), most β-catenin is localized in cell membrane along the length of the crypt. Whilst, E-cadherin is also localized in the cell membranes, expression is not uniform along the length of the crypt. Instead there are clusters of low E-cadherin expressing cells (forming “bands” or “rings” of green staining cells, see 3D panels Figure 5B) in the lower half of the crypt. There are also clusters of high E-cadherin cells at the base of the crypt, appearing as bright orange/yellow as they also express high levels of β-catenin (Figure 5B, RGB and 3D, yellow arrows). Of the 32 crypts scored, about 60% exhibited heterogeneous E-cadherin levels at the base of the crypts, i.e. low E-cadherin ‘bands’ of cells toward the bottom half of the crypt and high E-cadherin expression clusters of cells at the bottom and top half of the crypt. This clustering phenomenon of heterogeneous E-cadherin expression always occurs with high β-catenin levels. The remaining 40% of the crypts scored did not exhibit these clustering features (Figure 5C). We classified these two distinct crypt populations as heterogeneous (HE, 60%) and homogenous (HO, 40%) crypt populations.

Membrane localized E-cadherin and β-catenin expression also appears to exhibit different apical, basal and lateral distributions (Figure 5). E-cadherin localizes to lateral-apical (Figure 5B, red arrows) and basal (Figure 5B, blue arrows) membrane boundaries while β-catenin distributes along the lateral membrane (Figure 5B, red arrows) with some cells having basal or apical localization (Figure 5B, green arrows).

### 3D quantitation of subcellular localization of β-catenin and E-cadherin along the length of the crypt

**Figure 6:**
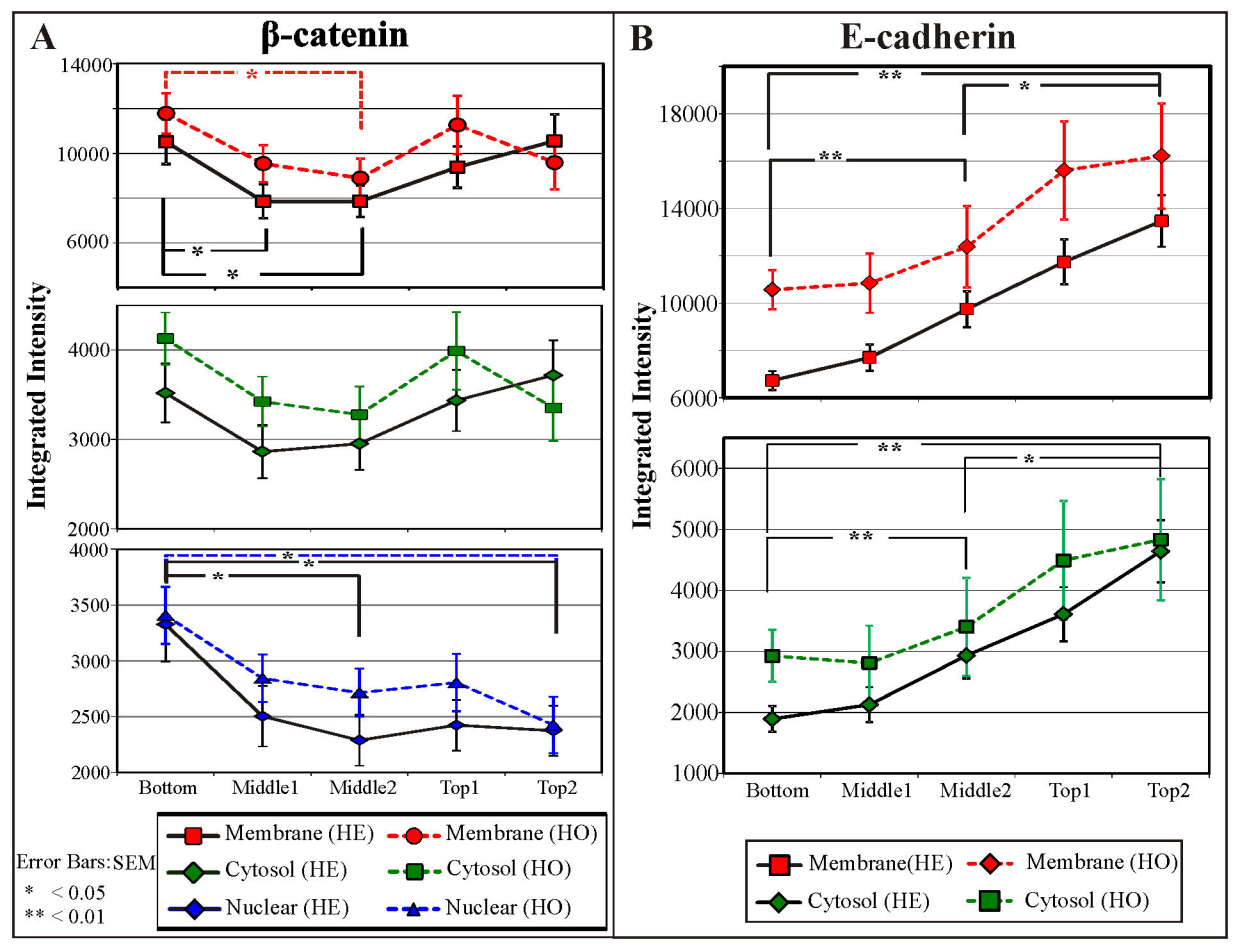
Subcellular distribution of β-catenin and E-cadherin the colon crypt compartments. Isolated crypts images were divided into five evenly divided regions (from the top of the crypt to the base, Figure 5B) with levels of β-catenin and E-cadherin quantified. The relative integrated intensities of β-catenin (A) and E-cadherin (B) in the nuclear, cytosol and membrane compartments were quantified for each region and crypt population. The E-cadherin integrated intensities were background subtracted with the nuclear intensities (no E-cadherin is expected in the nucleus) of the respective regions. Nuclear β-catenin is higher at the bottom of the crypt while the membrane compartments appear to be lower at the middle regions of the crypt. An increasing E-cadherin level was observed towards the top of the crypt for both the cytosol and membrane compartments. Note: 32 crypts were quantified (n=32, 20 HE-crypts and 12 HO-crypts). The error bars depict the RMS standard error of the mean (SEM).

Two length scales of compartmentalization were established, firstly at the whole crypt scale, crypt compartments (see Figure 5C RGB panel), and secondly at the individual cell scale, the subcellular compartments (the nucleus, cytosol and membrane sub-compartments, Figure S3). We quantitatively compared the β-catenin and E-cadherin immunofluorescence intensities in the different crypt compartments. Each crypt was considered in five evenly divided compartments, from the top to the bottom of each crypt: these compartments are labeled, bottom, middle1, middle2, top1 and top2 regions. The subcellular distribution (membrane, cytoplasmic and nucleus) of E-cadherin and β-catenin staining for each of the crypt compartments and for both HE and HO populations are shown in Figure 6A (β-catenin) and Figure 6B (E-cadherin). 

In all crypt compartments, β-catenin is primarily localized to the membrane compartment (Figure 6A, as observed in Figure 5B-C), but with significantly lower levels of β-catenin in the middle of the crypts. For the HE crypts, the difference in membrane β-catenin between the mid-crypt region and crypt base is more pronounced. Nuclear β-catenin is significantly higher at the crypt base (Figure 6A) compared to the mid-crypt region for HE crypts, but not for the HO crypts. This verifies our observation of higher nuclear β-catenin in some cells at the colon crypt base in Figure 5A (HE crypt). E-cadherin expression (Figure 6B) increases significantly from the base (low levels) to the top of the crypt in both the membrane and cytosol compartments for the HE crypt population. An increasing trend towards the top of the crypt is also observed for the HO crypt population. 

The HE crypt population (i.e. crypts with low E-cadherin cell clusters at the base) is characterized by high membrane and nuclear β-catenin levels at the crypt base (relative to mid-crypt). Heterogeneity in this crypt population is characterized by small clusters of high membrane E-cadherin and β-catenin expressing cells interspersed amongst the low E-cadherin expressing cell clusters (as observed in Figure 5B, yellow arrows). The HO crypts (i.e. crypts without the low E-cadherin cell clusters at the crypt base) do not exhibit these distinctive cell clusters and have higher membrane β-catenin at the crypt base compared to cells in the mid-crypt. E-cadherin expression increases as you move from the base toward the top of the crypts. 

### β-catenin and E-cadherin localization at sites of crypt budding

**Figure 7:**
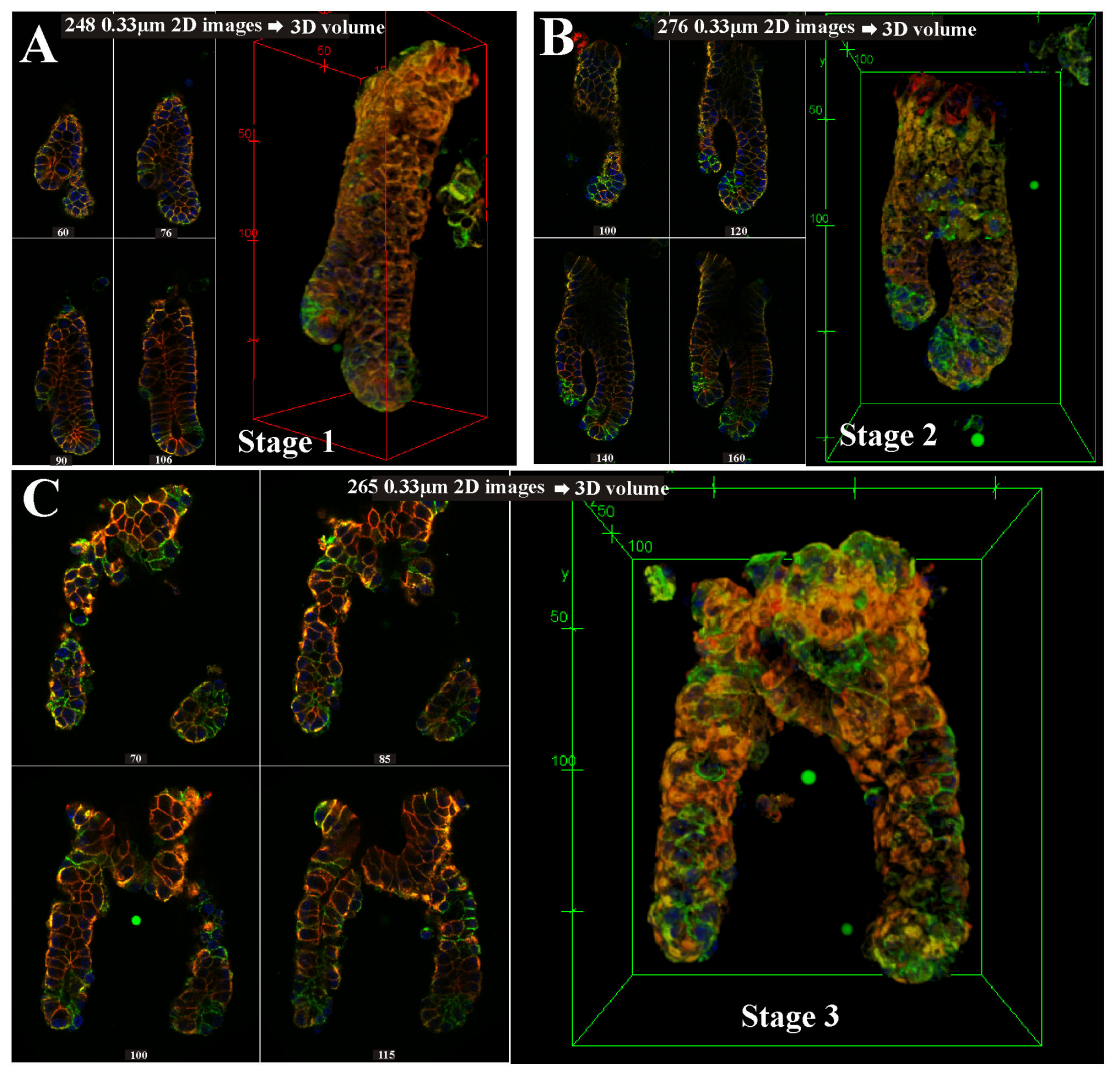
β-catenin and E-cadherin levels in budding colon crypts. Immunofluorescent 2D montage images extracted from 3D image stacks of isolated colonic crypts undergoing budding in (**A**) stage 1, (B) stage 2 and (**C**) stage 3 respectively, with the overlaid RGB, DAPI, β-catenin, E-cadherin images and 3D reconstruction shown. The image sequence indicates the daughter crypt extending from the bottom half of the parent crypt (stage 1) and extends upwards (stage 2) before gradually growing into mature crypts (stage 3). Non-uniformity in the β-catenin and E-cadherin staining can be observed (HE-crypt population) at localized regions near the base of the crypts. Crypt formation process is asymmetrical with the daughter crypt developing as a bud from the lower half of the parental crypt. A movie of the 3D budding crypts (stage 1) is available in the supporting information Video S1. Note: 3D images and movies were produced using Fiji/ImageJ 3D Viewer[68]. Intensity calibration microspheres are images together with the crypts for calibration and intensity standardization within and between images. Each image of the crypt is made up from more than 200 2D confocal images sections to generate the 3D reconstruction. Section numbers of 2D confocal images displayed in the individual montage are shown below each figure.

One of the key advantages of using the quantitative 3D confocal imaging technique is that subcellular protein levels can be analyzed during different stages of budding. Figure 7 shows confocal images and 3D volume reconstruction images of three isolated crypts at various stages of crypt budding. Figure 7A shows the image montage and 3D reconstruction of a colon crypt in stage 1. Asymmetrical bud formation is in progress with the daughter bud emerging near the base of the parent crypt. From the montage of 2D confocal sections, it can be seen that the small cluster of cells with high E-cadherin and β-catenin at the base of the crypt have a higher E-cadherin lateral-apical membrane expression, giving a bright red apical signal. Low E-cadherin cell clusters are observed near the base of the crypts and associated with the new bud. Figure 7B shows a stage 2 crypt, verifying the asymmetrical process with the daughter crypt clearly smaller than the parent. Again the high E-cadherin expression at the small cell cluster at the crypt base and the low E-cadherin expressing bands associated near the crypt base and new bud can be seen clearly. Figure 7C completes the proposed asymmetrical budding sequence with a pair of stage 3 crypts, showing the heterogeneity of the E-cadherin and β-catenin expressions.

The β-catenin and E-cadherin levels and spatial distribution at sites of crypt budding can be measured quantitatively (Figure 7) as clusters of cells (patches) with different graded color intensities. 3D animations of these budding crypts can be downloaded from the supporting information, Video S1. It is clear that at the interface between the daughter and parent crypt, there are patches of bright green fluorescence, i.e. high β-catenin (green), low E-cadherin (red). There are also clusters of low E-cadherin cells associated with the new bud. The distribution of E-cadherin and β-catenin is heterogeneous at the base of both the daughter and parent crypts. In this study, the proportion of budding crypts captured among the 32 whole crypts was too low for further analysis of the new buds. 

## Discussion

Two processes are responsible for cell production in the intestinal mucosa: continuous cell production, migration and turnover within a crypt (i.e. cell maintenance) and the production of new crypts (i.e. crypt production). 

### Intestinal crypt cell maintenance

Crypt progenitors in the transit amplifying region of the crypt divide every 12-16 hours producing over 300 cells per crypt every day [14]. These proliferating transit-amplifying (TA) cells form a continuous sheet [36,37] that moves up the crypt and into the villus walls. Up to six cell divisions are made as the cells migrate up the crypt [14] with a transit time from the bottom to the tip of the crypt of only 2-3 days [38,39]. As the TA cells move to the upper third of the crypt, proliferation reduces and differentiation increases, giving rise to differentiated cells belonging to two main classes, absorptive (enterocytes) and secretive (Goblet cells which secrete mucus and enteroendocrine) cells [40]. In the small intestine, Paneth cells are also produced, but these cells move to the bottom of the crypt [41,42]. Paneth cells are the only terminally differentiated cells at the bottom of the small intestinal crypts, but they are absent from the base of colon crypts [43,44]. To maintain cell numbers, the mature differentiated cells are removed from the tip of the villus in the small intestine (or from the surface epithelium in the large intestine) by exfoliation [45], apoptosis [46] and/or engulfment of effete colonocytes by phagocytes [47,48]. 

### Intestinal crypt production

Crypt production is most obvious in young animals; however, there is also a significant level of crypt production in normal adult intestinal mucosa. The production of new crypts has been termed crypt fission [15,16]. New crypt production decreases as animal age [15,19]. It was generally believed that fission of intestinal crypts is symmetrical [17,18], but our studies on the isolated crypts (see Figure 3 and Figure 4) indicate that new colon crypts can also be produced asymmetrically by budding from cells near the base of the crypt. It is interesting to note that two types of stem cells have been described in intestinal crypts, quiescent, LGR5 negative [5] and actively dividing, LGR5 positive cells (crypt base columnar) [12]. Perhaps they have different functions i.e. crypt maintenance or crypt production. 

### Signaling pathways regulating crypt production and implications for colorectal cancer

Wnt signaling has been linked with crypt production (fission) in infant rats [24] and is required for the production of colon crypts in mice *in vitro* [21,25]. Mutations in key components of the Wnt signaling pathway occur in colorectal cancer [49-52], implicating this pathway in colorectal cancer development. The cell-cell adhesion pathway is also linked to the Wnt pathway [53,54] with excess Wnt signaling leading to the loss of cell-cell adhesion junctions [55,56]. Restoration of fully functional APC is sufficient to restore cell-cell adhesion and to reduce the tumorigenic properties of colon cancer cell lines [57]. Understanding the relationship between excess Wnt signaling and cell-cell adhesion signaling is likely to aid development of targeted therapies against colon cancer.

### Crypt development and adenoma formation

Morphologically, normal colon crypts are close-packed, oriented with the crypt luminal axis perpendicular to the mucosal surface. Mutation of the APC gene causes abnormal crypt production, disorientation of the crypts, leading to colorectal adenomas [23]. Increased crypt production is the key feature driving the growth of these adenomas [23]. At present, it is not known how disordered crypt production occurs, nor is there a good understanding of underlying changes in biochemical signaling dynamics, either spatially or quantitatively, which would lead to crypt budding in adenomas (see Figure 1E and 1F in ref [23]). However, from the study by Wong et al [23] and an earlier study by Chang [58], it appears that adenoma formation is a perturbation of the normal crypt production processes rather than major changes in the processes maintaining cell production within a crypt.

From the confocal images of isolated crypts taken at different stages of development in this study, we predict a mechanism for normal crypt production which involves four stages (Figure 3). These and other observations made during morphological budding analysis prompt the theory that the normal crypt production cycle constraints the daughter crypt to reach stage 3 before any new budding can occur in the parent crypt (Figure 3). The resulting questions are, on what and how the underlying signaling pathways regulate the crypt budding cycle and constraint the rate of crypt production in the colon.

**Figure 8:**
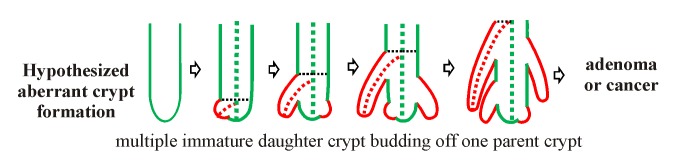
Schematic of hypothesized aberrant crypt budding. Perturbation of the normal crypt production sequence is hypothesized to lead to deregulated crypt budding where the parental crypt starts budding before the original daughter crypt has finished its crypt production sequence. This leads to multiple daughter crypts on one parent crypt consistent with the crypt budding seen on adenomas (see Figure 1E and 1G in ref [23]).

In adenoma, it can be hypothesized that aberrant signaling associated mutations in a key signaling pathway leads to a deregulation of crypt budding. Uncontrolled/un-constrained formation of new buds drastically changes the crypt morphology (Figure 8). This over-production of new crypts may explain the formation or initiation of adenomas (see Figure 1E and 1G in ref [23]) where multiple buds appear to be produced from a single crypt. The crypt isolation and 3D image analysis techniques described here could be applied to interrogate crypt production during adenoma formation (e.g. using APC^min/+^ [17] or APC^580S^ [59] mice).

Preliminary morphological studies of crypts isolated from APC^min/+^ and Tg(A33-CreERT2);Apc^fl/fl^ mice (24 hour Tamoxifen induction to induce truncation of APC) indicate no occurrence of multiple budding on a single crypt (not a surprising result considering no adenoma were present in the colons of these mice) (Figure S6). However crypts from Tg(A33-CreERT2);Apc^fl/fl^ mice did show differences in crypt basal lumen size, suggestive that APC truncation does alter crypt morphology (Figure S6A and B). The crypt budding stages observed in these mice are consistent with the four stages proposed in this study. These initial observations support the current findings although further experiments on crypts isolated from colon adenoma are required.

### Spatial cellular signaling and crypt formation

Wnt signaling and cell-cell adhesion have a role in crypt development [21,22,25,28,60]. Analysis of the regional and subcellular distribution of E-cadherin and β-catenin along the length of the crypt revealed two distinct populations of crypts. One population consists of crypts with a heterogeneous distribution of E-cadherin and β-catenin (HE-crypts) with specific clusters of low membrane E-cadherin cells residing just below the middle of the crypt. At the base of the crypt, low E-cadherin expressing cells are bordered by small clusters of high E-cadherin and β-catenin expressing cells. This HE crypt population makes up 60% of the crypts analyzed in this study. The other population of crypts is those having a more uniform β-catenin and E-cadherin distribution along the crypt length (HO-crypts). Interestingly, detailed 3D analysis of a series of budding crypts in the three stages of the crypt production cycle reveals similar E-cadherin low and β-catenin high patterning of cells at the junction and bases of the parent crypt and daughter crypt (Figure 7). These observations suggest there is a functional role for the heterogeneous patterning of Wnt and cell-cell adhesion components during crypt formation.

Clearly, the loss of E-cadherin could have a role in cell-cell localization and migration during the budding process. The low E-cadherin might allow cells to move into the trans-amplifying (TA) region of the crypt or to facilitate bud formation. The clusters of cells with high E-cadherin at the base of the crypt might have a structural role in ‘anchoring’ these cells to other cells at the base of the crypt. Another possibility is that cross-talk between the Wnt and other cell-cell adhesion signaling pathways [61] alters the spatial patterning and distribution of E-cadherin at the locality of bud formation in preparation for the emergence of the bud. The functional significance of the two distinct populations of crypts (HE and HO) and the E-cadherin high/low clusters need to be analyzed further by bringing together information on the functional and structure changes in colon crypts and new spatial biochemical data relevant to the underlying signaling activities at the onset of crypt production. 

### Understanding normal and aberrant crypt formation using colon crypt cultures

The technique presented in this study provides a platform to understand early steps in normal crypt development. Bringing together the findings in this study, it is clear that crypt production is a complex, highly synchronized process which is not yet well understood. By using an integrated ‘systems approach’ to examine and quantitate simultaneously the morphology and biochemistry of isolated crypts, this study suggests an alternative asymmetrical mechanism of crypt formation in normal mouse mucosa. Our results suggest that in normal crypts the asymmetrical crypt formation process is constrained so that only one new crypt is produced at a time (we did not see any normal crypts that had multiple buds). Reconciling the morphological differences between normal and adenomatous crypts (with multiple crypts forming off one crypt), it is reasonable to predict breakdown of this singular constrain, leading to multiple aberrant bud formation from each crypt during adenoma formation. To understand the origin of this single bud constraint mechanism, we have used high resolution 3D immunofluorescence data to delve into the biochemistry of the bud process. Here we have demonstrated heterogeneous (as opposed to homogenous) expression of Wnt and cell-cell adhesion target proteins (β-catenin and E-cadherin, both implicated in crypt production and cancer) at the focal junctions associated with asymmetrical budding. This heterogeneity in the distribution of Wnt and E-cadherin is likely to represent important markers of where the budding will occur (so giving clues at the budding constraint mechanism(s)). A systems understanding of how the underlying cellular signaling pathway influences crypt production is needed.

Clearly further work is required, but with this knowledge and these 3D-techniques, it will be possible to differentiate between normal and mutant (cancer-causing) crypt development and to further clarify the roles of signaling pathways in crypt production and initiation of colon cancer. Our laboratory has developed an enhanced co-culture system for producing cultured colon crypts in 4 to 6 days (significantly earlier than previously reported [62]). Using this technique we can monitor the dynamics of crypt formation and study murine colon crypt formation from diverse genetic backgrounds (e.g. APC^min/+^ [17] or APC^580S^ [59]). 

Further investigations into the specifics of crypt formation in terms of key signaling protein localization at particular spatial locations using this quantitative 3D imaging technique are on-going. Using live cell imaging, the growth of crypts in culture can be monitored to capture the process of crypt formation as well as determining the biochemical changes on the surface of the developing colon spheroids. These experiments will provide data for the sequence of molecular mechanisms controlling crypt formation in colon mucosa during normal homeostasis and adenoma formation.

### Quantitative computational modeling of crypt development

This data will provide critical insights for molecular and cancer biologist, it will also provide key parameters for the development of predictive cellular computational models of colon crypts. Mathematical models of colon crypts have emerged in recent years [63-67], attempting to provide a system view of the signaling networks and cellular morphology of colon crypts. These studies typically lack quantitative data for building or validating the models. By integrating the experimental observations and measurements with systems based computer modeling, development of predictive cellular models of colon crypts is now within reach. These models will be able to predict the molecular causes of aberrant colon crypt development resulting from mutations in APC.

### Systems approach to investigate crypt production across different length scales

In this study, we have used a 3D confocal imaging technique to investigate the distinctive morphology of isolated colonic crypts from different regions of the colon, establishing that crypt budding is one of the mechanisms responsible for the production of new crypts, characterizing a heterogeneous subcellular distribution of β-catenin and E-cadherin in a majority (60%) of the crypts analyzed and that this heterogeneity is present in budding crypts suggesting a possible link between development and the cellular signaling pathways.

The observations were possible due to the investigation of colonic crypts at different scales (i.e. from proximal, middle and distal regions of the colon, the crypt compartments and the subcellular compartments, see Figure 1). At the largest scale, investigating differences in morphology between crypts isolated from proximal, middle and distal regions of the colon; at the crypt level, where spatial and quantitative differences in signaling proteins (β-catenin and E-cadherin) distributed in different regions of the crypt; down to the smallest scale the subcellular level of proteins where quantitative localization of proteins (β-catenin and E-cadherin) between the membrane, cytoplasm and nucleus subcellular compartments, is critical for understanding the regulation of crypt production and maintenance. 

A systems approach to the investigation of colon mucosa will improve our understanding of the relationships between crypt formation and the maintenance of crypt cell production. Crypt isolation and the 3D imaging technique provide an excellent tool to generate morphological and biochemical spatial and dynamic data from different animal model systems and provide insights and quantitative data to facilitate more in-depth modeling of signaling pathways implicated in colon crypt development. 

## Materials and Methods

### Animals

C57BL/6 mice were maintained in the animal facility at Ludwig Institute for Cancer Research, Melbourne Parkville Branch. These mice were bred under specific pathogen-free conditions and all animal experiments were approved by the Ludwig Institute for Cancer Research/University of Melbourne Department of Surgery Animal Ethics Committee (approval number 002/11). APC^min/+^ and Tg(A33-CreERT2);Apc^fl/fl^ mice colon kindly provided by Dr Michael Buchert (The Walter and Eliza Hall Institute of Medical Research, Victoria, Australia).

### Antibodies and reagents

The antibodies used in this study were: rabbit anti-β-catenin (Sigma-Aldrich, Saint Louis, MI, USA, cat#C2206) and rat monoclonal anti-E-Cadherin (Invitrogen Inc, Camarillo, CA, USA, cat#13-1900, clone ECCD-2). The fluorescent stain 4, 6-diamidino-2-phenyl indole Nucleic Acid Stain (DAPI, cat# D1306 Molecular Probes Inc, Eugene, OR, Invitrogen) was used to stain the nuclei. The secondary fluorescent antibodies were Alexa Fluor^®^ 488 goat anti-rabbit IgG (H+L) (Molecular Probes Inc, Eugene, OR, Invitrogen cat#A-11034) and Alexa Fluor^®^ 546 goat anti-rat IgG (H+L) (Molecular Probes Inc, Eugene, OR, Invitrogen cat#A-11081).

### Isolation of intestinal crypts

Individual mouse intestinal crypts were isolated from the intestine of the mouse using a protocol similar to the method described by Whitehead et al 1999 [29] (Gastroenterology 117: 858-865). For adult mice (4 weeks and older), the proximal section was defined as 2 cm from the end of the caecum, the distal section was defined as 2 cm after the rectum. The mid-colon is defined as the portion in between proximal and distal sections (about 3cm for a 7 weeks old mouse). For young mice (younger than 4 weeks old), the colon was shorter than 2cm and therefore the colon (caecum to rectum) was divided equally into three sections (proximal, middle and distal). The whole colon or section was harvested, kept on ice and cut opened. The colon was rinsed in ice cold MT-PBS and treated with 0.04% (w/v) sodium hypochlorite in MT-PBS (20mM phosphate, 0.149M sodium chloride) for 5 minutes. After the sterilizing, the colonic mucosa was rinsed with MT-PBS and incubated for 90 minutes with 3mM EDTA and 0.05mM DTT in MT-PBS at room temperature. The mixture was allowed to settle under gravity and the supernatant discarded while the pellet was washed once with MT-PBS. 10mL of MT-PBS was added and the suspension shaken vigorously for 15 seconds to liberate the crypts from the sub-mucosa (into the crypt suspension supernatant). The crypt liberation process was repeated 5 times, consolidating the crypt suspension from each repetition. The crypt suspension was centrifuged at 400rpm (34g) for 5 minutes, discarding the supernatant and re-suspending the pellet in 3mL MT-PBS. The crypt suspension was then kept on ice. For crypt morphology and budding analysis, the protocol for the isolation is the same, except that for the proximal region of the colon (to take into account fragile nature of the crypts in that region), the incubation with 2mM EDTA was for a shorter time, 60 minutes instead of 90 minutes.

### Immunofluorescent staining of isolated crypts

Prepare an aliquot (1ml) of isolated crypt suspension. For fixation, gently mix in 25μL of 4% (v/v) paraformaldehyde (PFA, final concentration of 0.1% W/V) and incubate at 4°C for 48 hours. Centrifuge the suspension at 400rpm (34G) for 2 minutes. Discard supernatant fluid, re-suspend the pellet in 1ml of MT-PBS and repeat the centrifugation for 30 seconds. The supernatant was discarded and pellet re-suspended in 1ml of 0.2% (v/v) TritonX-100 in MT-PBS at room temperature for 15 minutes. Centrifuge the suspension at 400rpm (34G) for 30 seconds and discard supernatant fluid. Re-suspend the pellet in 1ml of 0.2% (w/v) Bovine serum albumin (BSA) in MT-PBS (to block non-specific antibody staining) and incubate at room temperature for 3 hours. Centrifuge the suspension at 400rpm (34G) for 30 seconds and discard the supernatant fluid.

The fixed and albumin blocked crypts were re-suspended in 100µl of primary antibody solution [0.2% BSA (w/v) in MT-PBS, rabbit anti-β-Catenin (Sigma-Aldrich #C2206, 1:400), and rat anti-E-Cadherin (Invitrogen #13-1900, 1:200)] and incubated at 4°C overnight. Gently mix 1ml of 0.2% (w/v) BSA in MT-PBS with the suspension and centrifuge for 30 seconds at 400 rpm (34G). The supernatant fluid is discarded, the pellet re-suspended in 1ml of 0.1% (v/v) TritonX-100 in MT-PBS and incubated for 5 minutes at room temperature. Centrifuge the suspension at 400 rpm (34G) for 30 seconds, discard the supernatant fluid and re-suspended the pellet in 1ml of 0.2% (w/v) BSA in MT-PBS, repeating the wash in BSA-PBS twice. The antibody staining procedure is then repeated for the secondary antibody with 100μL of secondary antibody solution (0.2% BSA (w/v) in MT-PBS) containing Alexa Fluor® 546 goat anti-rat IgG (1:400) and Alexa Fluor® 488 goat anti- rabbit IgG (1:400). For counter staining of the nucleus, the pellet is re-suspended in 200μL of 1nM DAPI in MT-PBS and incubated for 15 minutes at room temperature. 1ml of MT-PBS is added into the suspension and centrifuged at 400rpm (34G) for 30 seconds. The pellet contains the stained crypts which were re-suspended in 100μL of MT-PBS. 

To prepare for confocal imaging, an aliquot (15μL) of the stained crypts suspension is mixed with 20μL of Agarose mixture (1% w/v Agarose (Molecular Grade, cat# BIO-41025, Bioline, Luckenwalde, Germany), 0.25% w/v Bovine Serum Albumin in phosphate buffered saline kept at 55~60°C). A thin layer of the mixture was spread onto a glass coverslip (Menzel-Glaser, 25mm diameter, cat# CB00250RA1) presoaked in 90% (v/v) MeOH/DDW and air dried. The cover slip was inserted into a Sykes Moore Chamber (Bellco Glass Inc., Vineland, NJ) base followed by the Sykes Moore Gasket. An agarose “sandwich” was then constructed by adding 0.3ml of the Agarose mixture onto the cover slip, filling up the chamber with 0.2-0.3ml of MT-PBS and covering with another cover slip before completing the assembly of the chamber with the holder. The crypt preparation was stored in the dark at 4°C. Note that for crypt morphology and budding analysis, only the DAPI counter staining of the nucleus was necessary with the same mounting procedure as described above.

### 3D confocal fluorescence imaging

Immunofluorescent staining of the crypts was detected with an Olympus FV1000 Spectral Confocal attachment to an Olympus IX-81 microscope on either a 20x (for morphological and budding analysis) or a 60x water immersion lens (for spatial analysis of proteins). The crypts were imaged using standard filter sets and laser lines, acquiring single labeled images. DAPI, β-catenin and E-cadherin fluorescence were excited with the 405nm, 488nm and 546nm laser lines, respectively and the emission wavelength were measured at wavelengths 405nm, 473nm and 559nm, respectively. The images were captured using Olympus FluoroView software (Version 1.7c). 3D image stacks were acquired which encompasses the entire depth of the crypt(s) in the field of view. 

For crypt morphology and budding analysis, only the 405nm laser line (DAPI signal) and the phase contrast attachment was utilized to acquire data. The DAPI signal and the phase contrast signal was acquired using a motorized staged to gain multiple fields of views for each sample. The entire depth of the sample was acquired as 3D image stacks at approximately 20µm thickness for each optical section. For quantitative spatial analysis of key proteins in isolated crypts, cubic voxels were acquired for each image stack. The output analogue signal, representing the fluorescence intensities was digitized to 16 bits resolution at 65536 levels of grey and saved as an Olympus Image Binary (OIB) image.

### Colonic crypt morphological and budding analysis

The image files were imported into the ImageJ software (created by Wayne Rasband at the US National Institutes of Health [68]) for analysis. For each confocal 3D image stack (20x magnification, phase contrast and DAPI fluorescent), the isolated crypts were identified visually and measured by drawing segmented lines using the imaging tools of ImageJ/Fiji software (as shown in Figure 2C). Measurements were recorded using the ‘ROI Manager’ (Region Of Interest Manager) tools and exported as Microsoft Excel compatible worksheets. The length of each crypt is tabulated and analyzed in Excel. Single factor analysis of variance (ANOVA) test was applied to test for significant differences in the length and basal widths of different colon regions (i.e. proximal, middle and distal).

### Spatial compartment analysis of β-catenin and E-cadherin

The Olympus Image Binary (OIB) image files from the fluorescently stained individual whole mount crypts were imported into the ImageJ/Fiji software[68] for processing. 3D image stacks of each region were extracted and exported as ‘tiff’ files. The tiff files were then imported into MATLAB [69] for analysis. For cellular compartment analyses, the image stacks were processed based on the procedures outlined in Text S1, Figure S1 and Figure S2. The signal intensities for β-catenin and E-cadherin in the nucleus, cytosol and membrane compartments were quantified (Figure S4) and tabulated accordingly. For crypt regional analysis, each 3D crypt image was evenly divided along the length of the crypt from the top to the crypt base. 5 regions (as shown in Figure S3A, Figure 5B) demarcating the bottom (bottom), lower middle (middle1), upper middle (middle2), lower top (top1), upper top (top2) part of the crypt were obtained. Single factor analysis of variance (ANOVA) test was applied to test for significant differences in protein staining intensities for different crypt regions.

## Supporting Information

Text S1 Image processing and compartmental analysis.
(PDF)

Text S2 **Comparison of isolated crypt analyses with histological sectioning data.**
(PDF)

Figure S1 **Cellular compartment mask generation using image processing and segmentation.** (A) The multichannel 3D confocal image stacks are separated into individual channels, namely β-catenin, E-cadherin and DAPI. (B) Segmentation and binary operations are applied to individual channels to generate appropriate functional overlays/masks to be used for generating the compartment masks. One such operation shown here is an “OR” operation between E-cadherin and β-catenin followed by a “NOT” operation with the DAPI signal. (C) 3D compartment masks generated from the overlays (2D section shown). (D) The compartment masks are applied onto the 3D β-catenin and E-cadherin image stacks to analyze the intensity encompassed within the respective compartments.
(TIF)

Figure S2 **Schematic representation of image processing and segmentation.** (A) Each 3D multi-channel image is separated into the constitute channel image stacks, namely β-catenin, E-cadherin and DAPI. (B) The DAPI nuclear mask was obtained from the segmentation of DAPI signal image stack. (C) An “OR” operation is applied between β-catenin and E-cadherin signal stacks to obtain an enhanced membrane signal stack. Segmentation of the enhanced membrane stack yields the membrane mask with the final membrane mask obtained by excluding any nuclear noise apply a “NOT” operation with the DAPI nuclear mask. (D) The whole cell mask was obtained by applying an “OR” operation between the nuclear mask and a segmented E-cadherin signal stack (E-cadherin membrane mask) before filling in the hole of the resultant image stack. (E) The cytosol mask was determined by excluding the nucleus and membrane masks from the whole cell mask.
(TIF)

Figure S3 **3D subcellular compartmental quantitation of β-catenin and E-cadherin in the cells of colon crypts.** Isolated crypts image stacks were categorized into the 5 evenly divided regions of interest (A) and 3 sub-cellular compartments (nuclear, cytosol and membrane). Computational image analysis uses compartment masks for the whole 3D image stack of each crypt with the overlaying of the nuclear, cytosol and membrane masks onto the representative 2D intensity images of DAPI (B), β-catenin (C) and E-cadherin (D). The borders of the respective masks are marked with blue (nuclear), green (cytosol) and red (membrane). The signal intensities for each compartment through the 3D image stack was summed up and integrated with results shown in Figure S4.
(TIF)

Figure S4 **3D compartment Intensity Projections of β-catenin and E-cadherin in the colon crypt image stacks.** The intensity of the target protein β-catenin and E-cadherin in the respective compartments of the isolated colonic crypts are shown here as 2D intensity projection maps of the 3D image stack. The projected signal intensity map of each target protein is obtained by summing and then averaging up the 2D intensities through the depth of the image to give a 2D representation of the signal intensity of the 3D crypt. Data is presented here for two crypts (A and B). For each dataset, the top panels represent the intensity projection for β-catenin in the cytosol, membrane, nucleus and non-nuclear (cytosol + membrane) compartments while the lower panels represent E-cadherin staining. Panel A is a typical HE crypt with the presence of the E-cadherin high clusters at the bottom surrounded by a band of low E-cadherin expressing cells (about 60% of the analyzed population n=32) while panel B represents a typical HO crypt without the clear E-cadherin low clustering. It can be observed from the two analyses that with the E-cadherin high cluster, the surrounding low E-cadherin expression band has a correspondingly higher nuclear β-catenin expression. This is not the case for the crypts without the clusters of E-cadherin low cells. NOTE: The intensity projection images digitally scaled for display purposes [maximum intensity: A(β-catenin) 8800 of 65535, A(E-cadherin) 15800 of 65535, B(β-catenin) 8600 of 65535, B(E-cadherin) 16400 of 65535] and in particular the E-cadherin nuclear signal is scale down about 4x. The intensity projection of E-cadherin in the nuclear compartment is almost non-existent (very low), which is consistent with the notion that there is unlikely to be E-cadherin in the nucleus (extracellular domain targeted by antibody).
(TIF)

Figure S5 **Colon crypt lengths measured in stained tissue sections and isolated crypts.** Images of HE (Hematoxylin and Eosin) stained tissue samples for the proximal (A), middle (B) and distal (C) regions of the colon. Estimation of crypt length conducted by selection of whole crypt visible (most of the crypts are partially obscured due to sectioning, as indicated by arrows in C) in the image is shown by the green line segments. (D) HE estimated average crypt length is in the same range as that measured by crypt isolation and morphological analysis in this study. Note: Mean number of crypts scored are in brackets, images (A-C) shown are of the same magnification and the error bars are standard deviations.
(TIF)

Figure S6 **Morphology and β-catenin/E-cadherin distribution of isolated crypts from APC^min/+^ and Tg(A33-CreERT2);Apc^fl/fl^ mice.** Confocal images of crypts isolated from Tg(A33-CreERT2);Apc^fl/fl^ (A, B, F and G) and APC^min/+^ (C-E, I-J; 24h induction with Tamoxifen to induce APC truncation) mice. No aberrant crypts with multiple crypt buds were identified, however, the heterogeneity of β-catenin and E-cadherin distribution was also observed in these crypts. Crypt shape appears similar to normal C57BL/6 mice with the exception of the Tg(A33-CreERT2);Apc^fl/fl^ crypts which have an enlarged basal lumen (see A and B). Note: (A-E) Immunofluorescent confocal composite images for DAPI (blue), β-catenin (green) and E-cadherin (red), (F-G) confocal composite phase contrast image with DAPI. 3D volumetric reconstructions are shown in C and E. Scale bar: 30µm.
(TIF)

Video S1 **3D animation of a rotating 3D budding crypt.** A multichannel 3D confocal image stack (DAPI in blue, β-catenin in green and E-cadherin in red) of a crypt undergoing budding was rotated along the vertical axis of the image stack showing the distinct clusters of E-cadherin low expressing cells (highlighted in green) at the base of both the parent and daughter crypts. Movie created using Fiji/ImageJ 3D Viewer [68].
(AVI)
