# Supplementary material for: Colon Cryptogenesis: Asymmetric Budding
Source: PLoS One. 2013 Oct 21;8(10):e78519. doi: 10.1371/journal.pone.0078519 (PMC3804607; doi:10.1371/journal.pone.0078519)
Supplement: Text S1 — Image processing and compartmental analysis. (PDF) [file pone.0078519.s001.pdf]

Text S1: Image processing and compartmental analysis.

The image stacks acquired from the confocal microscope are in the Olympus Image Binary (OIB) format. This was converted to a portable tiff format readable by MATLAB [1] using Fiji/ImageJ [2] with the Bio-Format plugin. Import of 3D image stacks: Individual region 3D stacks of the regions defined in Figure S3A were also extracted using the drawing tools in Fiji/ImageJ. The extracted regional image stacks were saved as separate tiff files. Both the whole and region tiff files were imported into MATLAB for processing and analysis.

Image processing (Import and pre-processing): For every tiff file (both regions and whole crypt), the intensities for the different fluorescence channels were separated as shown in Figure S1 (Step A). In this study, DAPI (blue), β-catenin (green) and E-cadherin (red) channels were separated into their respective mono-colour stacks (Figure S1 Step A, Figure S2A and Figure 5). For each channel (mono-colour) signal stack, selective filtering and image intensity threshold were applied and the region of interest isolated. Specifically, DAPI marked the nuclei compartment, E-cadherin outlined the membrane boundary and β-catenin marked primarily the membrane and cytosol. 3D binary masks were generated for each signal (Figure S1, Step C).

Image processing (Compartment Masks): To generate the compartment masks, mathematical operations were applied to the signal intensities tiffs/masks. Three compartments (nuclear, cytosol and membrane compartments) were defined and used in this study. The nuclear mask was generated from the DAPI signal (Figure S2B). As observed from the individual signal images (Figure 5), both E-cadherin and β-catenin were primarily localised to the membrane. Taking into account the heterogeneity observed in the E-cadherin staining, in order to enhance the membrane signal's contrast and continuity, an "OR" operation was applied between the E-cadherin and β-catenin intensities image stack (see Figure S1, Step B and Figure S2C) and segmented to acquire an enhanced membrane mask. A "NOT" operation was applied between enhanced membrane mask and the nuclear mask to determine the final membrane mask (Figure S2C). A whole cell mask was generated using the DAPI nuclear and E-cadherin membrane masks (Figure S2D) and the cytosol mask was formulated from the difference between the whole cell mask, the nucleus and membrane masks (Figure S2E).

Image processing and Analysis (Signal Compartmentalisation): The 3D binary compartment masks (membrane, cytosol and nuclear, Figure S1 Step C) were applied over the β-catenin and E-cadherin 3D signal channels to quantify the expression and localisation of the signal

intensities in the respective compartments (Figure S1 Step D, Figure S3B-D). The total 3D intensities of signal residing in each compartment were quantified (Figure S4) and tabulated, together with the volume of each compartment (in the form of total pixels/voxels). These intensity and volume data were consolidated for all region image stacks. The integrated (relative) intensities per pixel for each crypt, compartment and region of the crypt were calculated and analysed statistically. These data provided compartment proportion information for each region as well as the change in intensities both in terms of subcellular compartments and regions along the length of the crypt.

References:

1. Mathworks (2007) MATLAB. version. 7.4.0.287 (R2007a) ed: The Mathworks, Inc.

2. Abramoff MD (2004) Image processing with ImageJ. Biophotonics Int 11: 36-42.
