# Supplementary material for: Colon Cryptogenesis: Asymmetric Budding
Source: PLoS One. 2013 Oct 21;8(10):e78519. doi: 10.1371/journal.pone.0078519 (PMC3804607; doi:10.1371/journal.pone.0078519)
Supplement: Text S2 — Comparison of isolated crypt analyses with histological sectioning data. (PDF) [file pone.0078519.s002.pdf]

Text S2: Comparison of isolated crypt analyses with histological sectioning data.

This crypt isolation and 3D imaging has an advantage over histological sectioning in that each crypt can be scored in 3 dimensions instead of one plane. It further improved the speed, efficiency and resolution of scoring the budding crypts when compared to micro-dissection [1,2,3] or serial sectioning [4]. The thickness of paraffin-embedded or frozen tissue sections is not accurate due to rough surfaces, undulations and inclinations with standard errors of up to 10% [5]. This causes inaccuracy in serial reconstruction. The accuracy of reconstruction is greatly improved using confocal sectioning as the slices are continuous. The removal of the mucosa layer from the crypt also enables direct identification of crypt surface and shape. These advantages allow finer details (i.e. stage 1 onset of fission process) of the budding process previously hidden among the sectioning gaps to be identified. From the data presented here, the colonic crypt production is primarily due to asymmetrical budding rather than symmetrical crypt fission [2,6].

As the animal ages, the budding frequency decreases and crypt production slows to a rate sufficient for maintenance and turnover (to maintain a stable crypt number). It is still unclear whether new crypt production is required to replace crypts lost through mechanical damage or whether all crypts have a definite lifetime. From our studies, the length of crypts from the proximal regions is shorter than the other regions of the colon. This observation is in agreement with Wright and Alison [7] who documented a smaller number of cells in proximal colon crypt. After birth, fission rate is high to populate the growing colon with sufficient crypts.

In early 1980s, Bjerknes and Cheng [8] measured Swiss albino mice colon crypt from perfusion isolated epithelium, estimating the crypt length to be 275±112 µm (SD, n=36) while Goodlad and Wright [9] measured the crypts from Balb-c mice using microdissection, estimating an average crypt length of 282.9±96.0µm (S.D, n=45) and basal crypt width of 44µm (calculated from data reported in ref [9]). They measured crypts from the whole colon without distinguishing the regions (proximal, middle and distal). The width reported by Goodlad and Wright is in range with the measurements made in this study (i.e. basal width of 42±7µm for 40 weeks old C57BL/6) although the average crypt length (156±30µm, 40 weeks old, all regions) reported here is shorter than both Goodlad and Wright [9] and Bjerknes and Cheng [8].

To make sense of the differences in crypt length reported, firstly it should be taken into account the different mouse strains used; Swiss albino mice are significantly larger in size than C57BL/6. Secondly during microdissection, primarily the large prominent crypts are scored (i.e. smaller crypts might be missed out) due to the low sampling numbers. Furthermore, in the 1980s, Goodlad and Wright used a visual measurement technique with a calibrated eyepiece graticule attached to the microscope while Bjerknes and Cheng measured crypt lengths off a 100x magnified image projection on a screen. We believe that the change from manual to digital measuring techniques may account for the differences in crypt length reported then and in this study. With the advancement of optical imaging, accurate digitised measurement is now possible. In this study, over two thousand individual crypts were isolated and scored for a population mean of crypt lengths in the colon using high resolution quantitative confocal microscopy measurement. The measurements obtained have substantially smaller variations than previously described (SD of 30µm compared to 96 and 112 µm). We also distinguish between the different regions of the colon (i.e. proximal, middle and distal) which have been shown to have significantly different in crypt lengths (Figure 2C).

To further validate the accuracy of the crypt length measurements used in this study, we compared crypt lengths estimated from sectioned samples from the proximal, middle and distal regions of the colon from C57BL/6 mice. Sectioning results were in a similar range to that reported in this study using isolated crypts confocal microscopy technique (Figure S5). It is also clear from Figure S5 that only limited number of whole crypts can be scored in each section due to obscuration from view of portions of crypts due to sectioning (white arrows). This evidence supports the notion that the crypt isolation technique described in this study is a more efficient and accurate method for studying crypt morphology.

This isolation method was particularly appropriate for colon as it preserves the whole crypt. In the small intestine, the villi are usually lost along with the top portions of the isolated crypts, making crypt identification more difficult. The crypts from the proximal regions of the colon tended to be more fragile and a different preparation (see material and methods section) is required to isolate intact proximal crypts.

References:

1. Dehmer JJ, Garrison AP, Speck KE, Dekaney CM, Van Landeghem L, et al. (2011) Expansion of Intestinal Epithelial Stem Cells during Murine Development. PLoS One 6: e27070.
2. Wasan HS, Park H-S, Liu KC, Mandir NK, Winnett A, et al. (1998) APC in the regulation of intestinal crypt fission. The Journal of Pathology 185: 246-255.
3. Park H-S, Goodlad RA, Wright NA (1995) Crypt Fission in the Small Intestine and Colon: A Mechanism for the Emergence of G6PD Locus-Mutated Crypts after Treatment with Mutagens. American Journal of Pathology 147: 1416-1427.
4. Bashir O, FitzGerald AJ, Goodlad RA (2004) Both suboptimal and elevated vitamin intake increase intestinal neoplasia and alter crypt fission in the ApcMin/+ mouse. Carcinogenesis 25: 1507-1515.
5. Masuda T, Kawaguchi J, Oikawa H, Yashima A, Suzuiki K, et al. (1998) How thick are the paraffin-embedded tissue sections routinely prepared in laboratory? A morphometric study using a confocal laser scanning microscope. Pathology International 48: 179-183.
6. Totafurno J, Bjerknes M, Cheng H (1987) The crypt cycle. Crypt and villus production in the adult intestinal epithelium. Biophysical Journal 52: 279-294.
7. Wright NA, Alison M (1984) The biology of epithelial cell populations. Oxford: Clarendon Press.
8. Bjerknes M, Cheng H (1981) Methods for the isolation of intact epithelium from the mouse intestine. The Anatomical Record 199: 565-574.
9. Goodlad RA, Wright NA (1983) Effects of addition of kaolin or cellulose to an elemental diet on intestinal cell proliferation in the mouse. British Journal of Nutrition 50: 91-98.
