# Supplementary figures and images for: Colon Cryptogenesis: Asymmetric Budding
Source: PLoS One. 2013 Oct 21;8(10):e78519. doi: 10.1371/journal.pone.0078519 (PMC3804607; doi:10.1371/journal.pone.0078519)

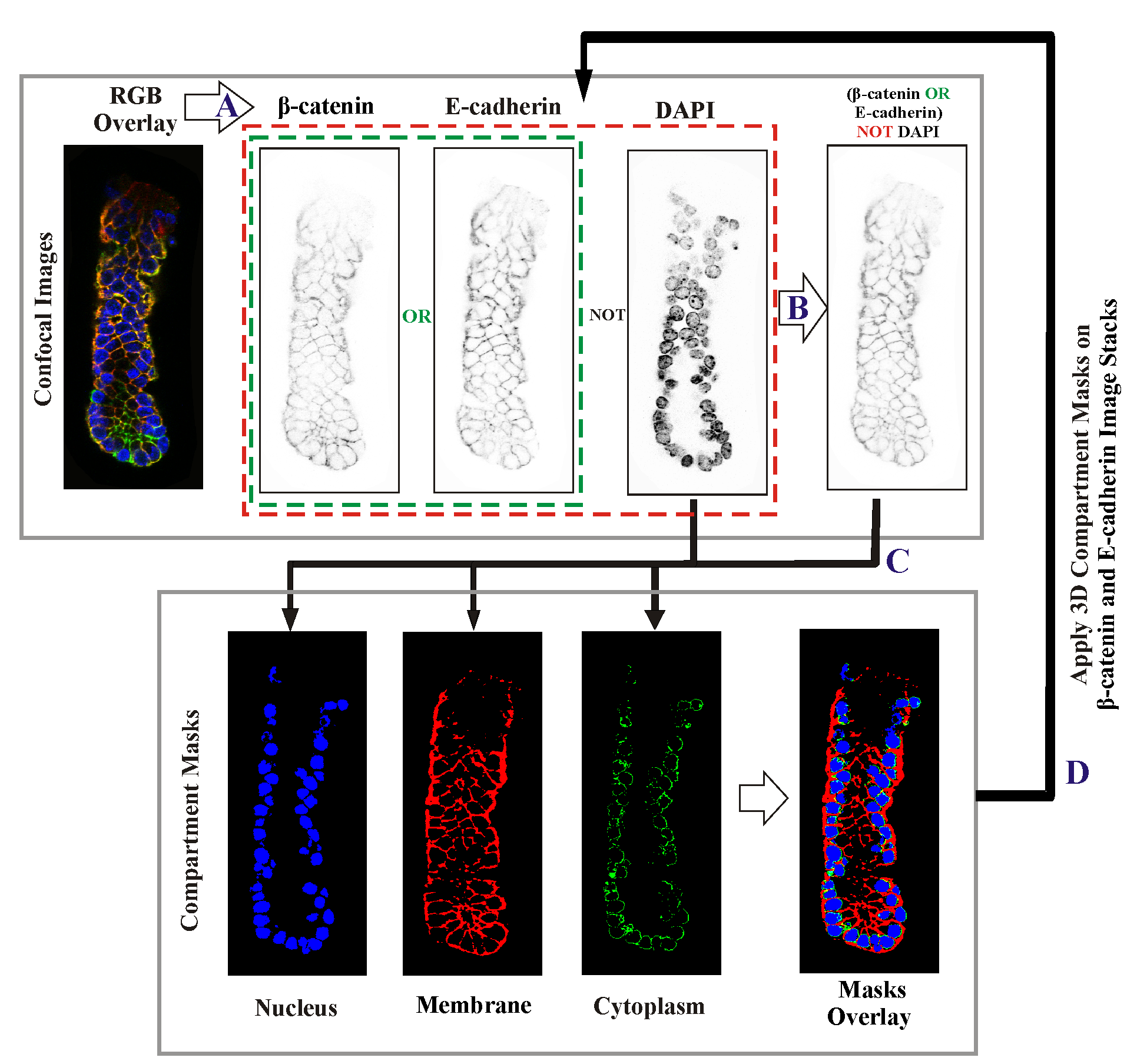

Supplement: Figure S1 — Cellular compartment mask generation using image processing and segmentation. (A) The multichannel 3D confocal image stacks are separated into individual channels, namely β-catenin, E-cadherin and DAPI. (B) Segmentation and binary operations are applied to individual channels to generate appropriate functional overlays/masks to be used for generating the compartment masks. One such operation shown here is an “OR” operation between E-cadherin and β-catenin followed by a “NOT” operation with the DAPI signal. (C) 3D compartment masks generated from the overlays (2D section shown). (D) The compartment masks are applied onto the 3D β-catenin and E-cadherin image stacks to analyze the intensity encompassed within the respective compartments. (TIF) [file pone.0078519.s003.tif]

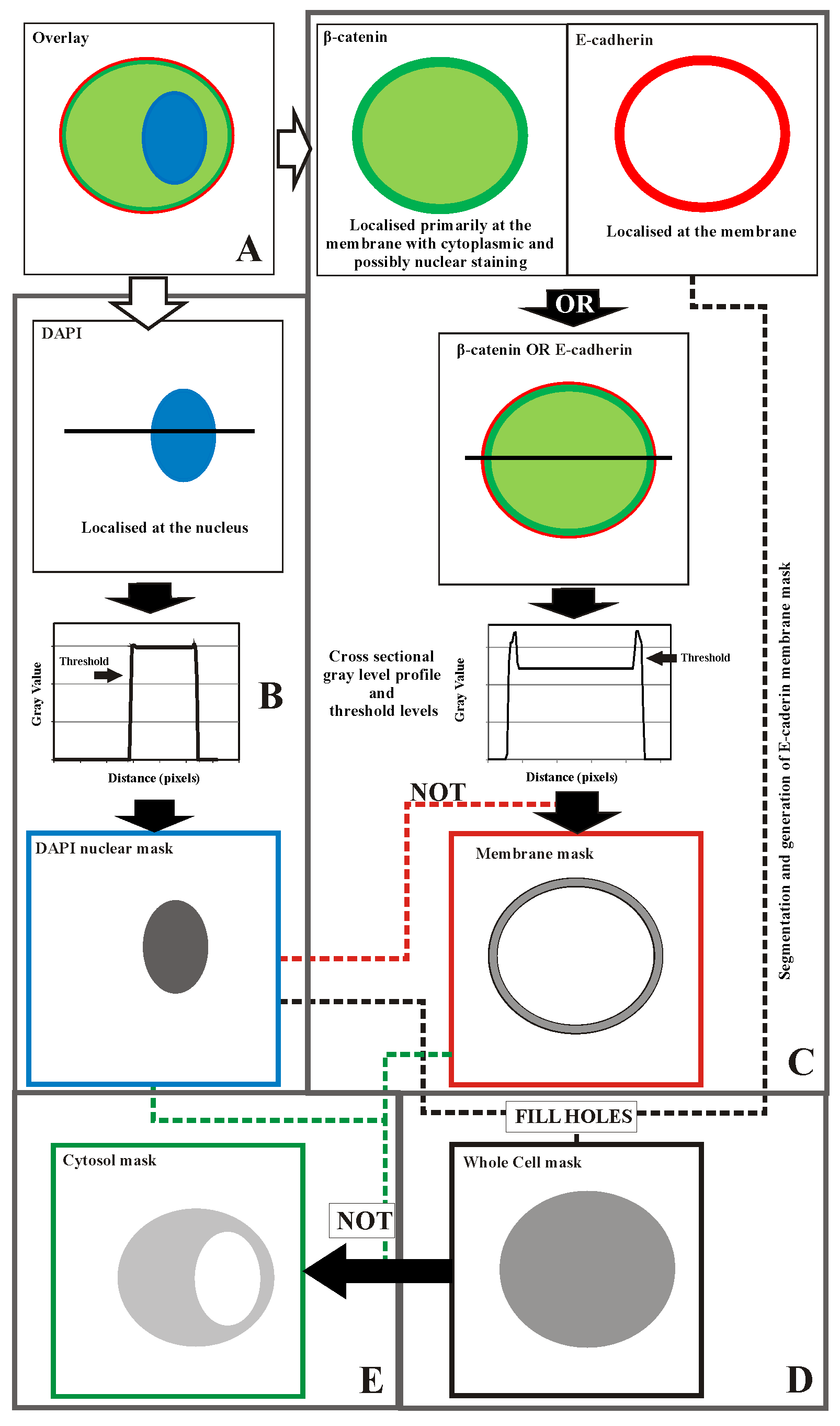

Supplement: Figure S2 — Schematic representation of image processing and segmentation. (A) Each 3D multi-channel image is separated into the constitute channel image stacks, namely β-catenin, E-cadherin and DAPI. (B) The DAPI nuclear mask was obtained from the segmentation of DAPI signal image stack. (C) An “OR” operation is applied between β-catenin and E-cadherin signal stacks to obtain an enhanced membrane signal stack. Segmentation of the enhanced membrane stack yields the membrane mask with the final membrane mask obtained by excluding any nuclear noise apply a “NOT” operation with the DAPI nuclear mask. (D) The whole cell mask was obtained by applying an “OR” operation between the nuclear mask and a segmented E-cadherin signal stack (E-cadherin membrane mask) before filling in the hole of the resultant image stack. (E) The cytosol mask was determined by excluding the nucleus and membrane masks from the whole cell mask. (TIF) [file pone.0078519.s004.tif]

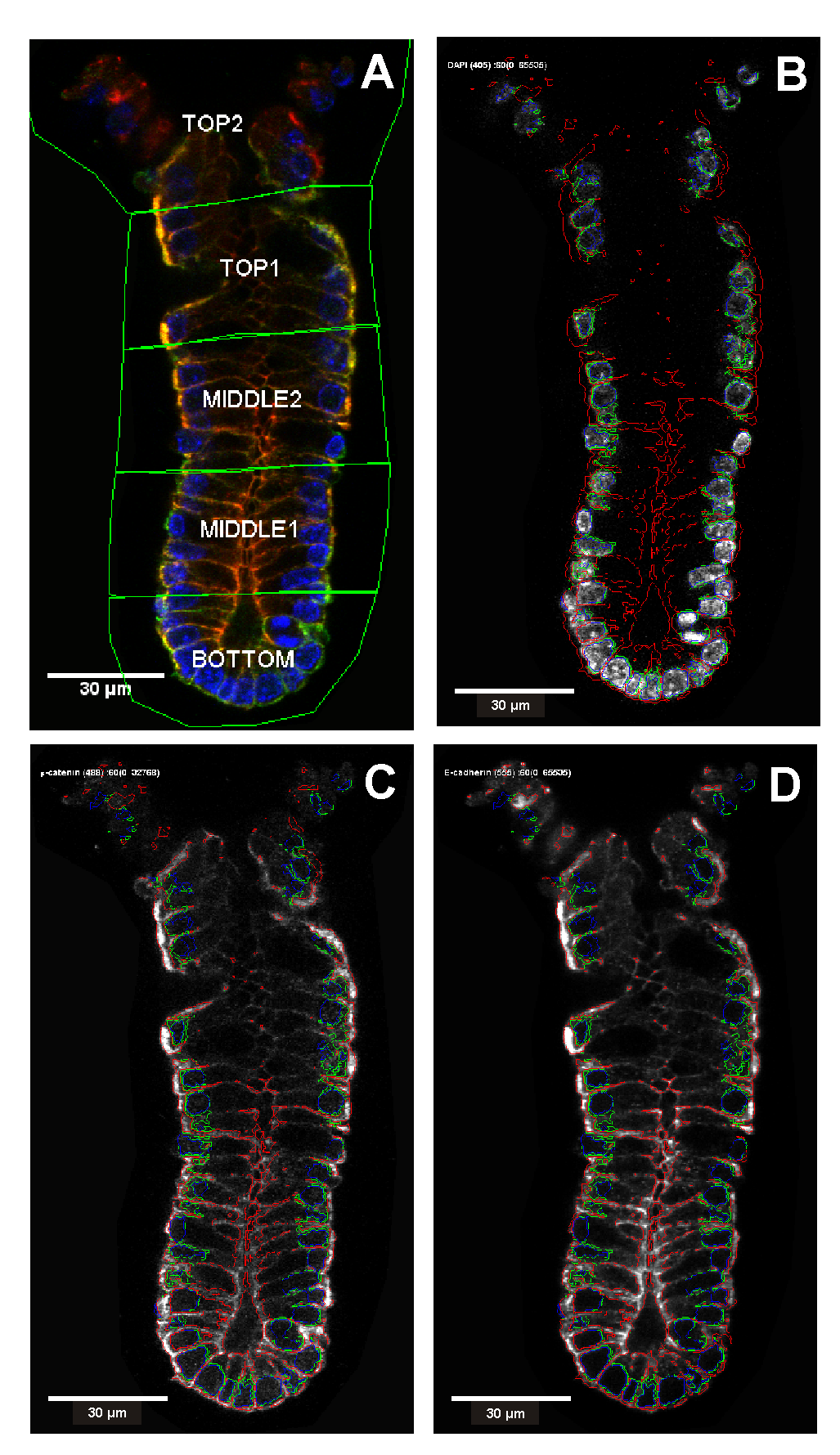

Supplement: Figure S3 — 3D subcellular compartmental quantitation of β-catenin and E-cadherin in the cells of colon crypts. Isolated crypts image stacks were categorized into the 5 evenly divided regions of interest (A) and 3 sub-cellular compartments (nuclear, cytosol and membrane). Computational image analysis uses compartment masks for the whole 3D image stack of each crypt with the overlaying of the nuclear, cytosol and membrane masks onto the representative 2D intensity images of DAPI (B), β-catenin (C) and E-cadherin (D). The borders of the respective masks are marked with blue (nuclear), green (cytosol) and red (membrane). The signal intensities for each compartment through the 3D image stack was summed up and integrated with results shown in Figure S4. (TIF) [file pone.0078519.s005.tif]

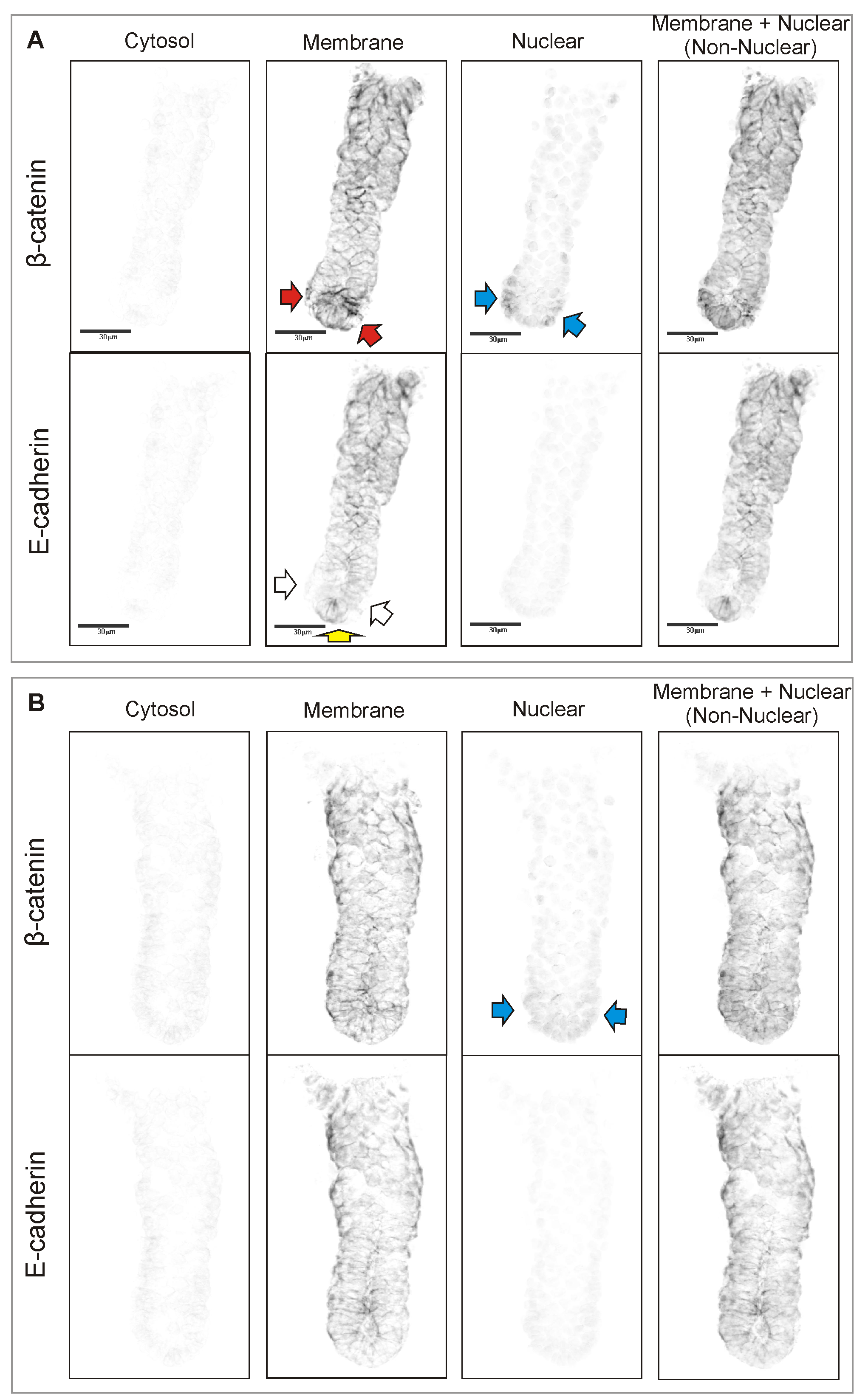

Supplement: Figure S4 — 3D compartment Intensity Projections of β-catenin and E-cadherin in the colon crypt image stacks. The intensity of the target protein β-catenin and E-cadherin in the respective compartments of the isolated colonic crypts are shown here as 2D intensity projection maps of the 3D image stack. The projected signal intensity map of each target protein is obtained by summing and then averaging up the 2D intensities through the depth of the image to give a 2D representation of the signal intensity of the 3D crypt. Data is presented here for two crypts (A and B). For each dataset, the top panels represent the intensity projection for β-catenin in the cytosol, membrane, nucleus and non-nuclear (cytosol + membrane) compartments while the lower panels represent E-cadherin staining. Panel A is a typical HE crypt with the presence of the E-cadherin high clusters at the bottom surrounded by a band of low E-cadherin expressing cells (about 60% of the analyzed population n=32) while panel B represents a typical HO crypt without the clear E-cadherin low clustering. It can be observed from the two analyses that with the E-cadherin high cluster, the surrounding low E-cadherin expression band has a correspondingly higher nuclear β-catenin expression. This is not the case for the crypts without the clusters of E-cadherin low cells. NOTE: The intensity projection images digitally scaled for display purposes [maximum intensity: A(β-catenin) 8800 of 65535, A(E-cadherin) 15800 of 65535, B(β-catenin) 8600 of 65535, B(E-cadherin) 16400 of 65535] and in particular the E-cadherin nuclear signal is scale down about 4x. The intensity projection of E-cadherin in the nuclear compartment is almost non-existent (very low), which is consistent with the notion that there is unlikely to be E-cadherin in the nucleus (extracellular domain targeted by antibody). (TIF) [file pone.0078519.s006.tif]

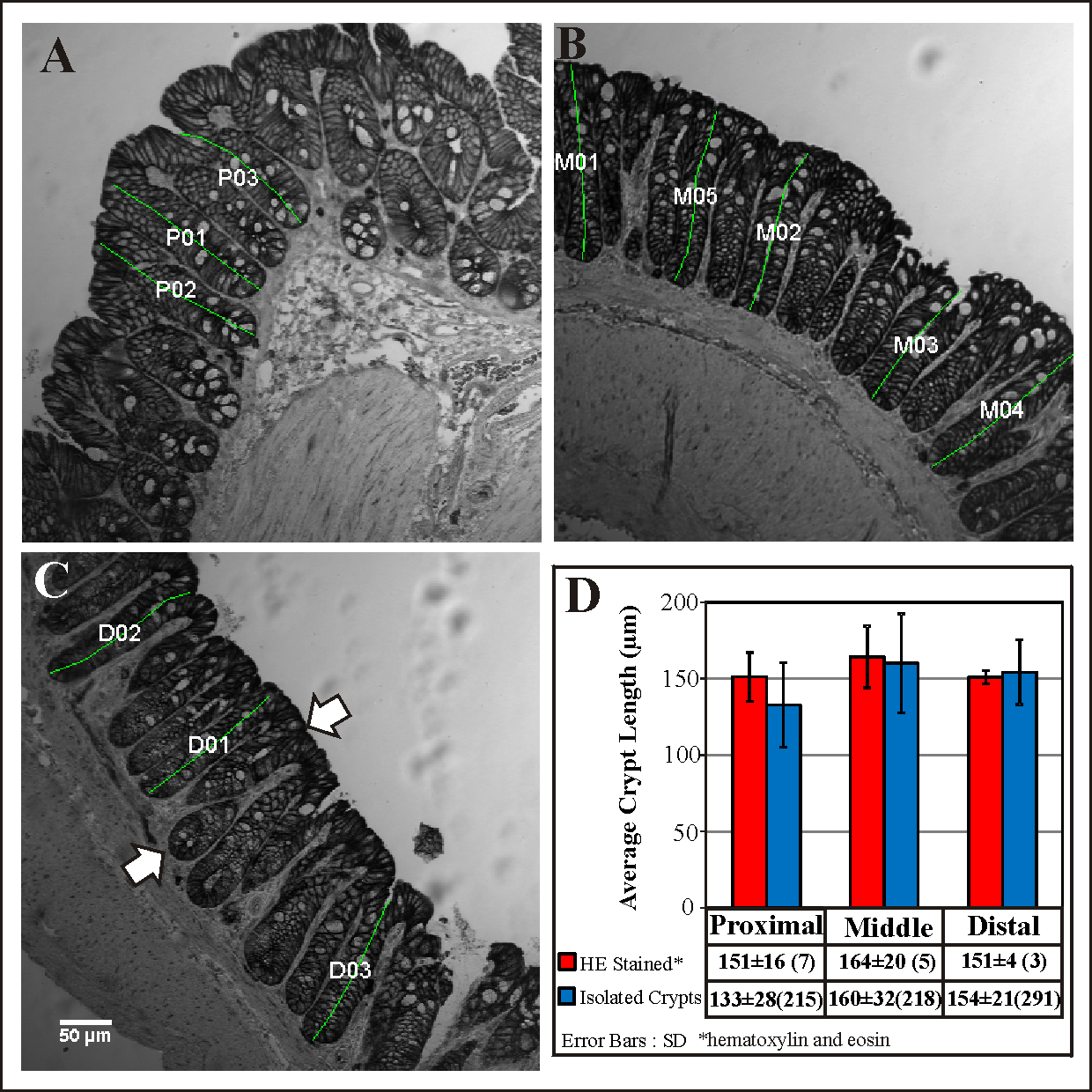

Supplement: Figure S5 — Colon crypt lengths measured in stained tissue sections and isolated crypts. Images of HE (Hematoxylin and Eosin) stained tissue samples for the proximal (A), middle (B) and distal (C) regions of the colon. Estimation of crypt length conducted by selection of whole crypt visible (most of the crypts are partially obscured due to sectioning, as indicated by arrows in C) in the image is shown by the green line segments. (D) HE estimated average crypt length is in the same range as that measured by crypt isolation and morphological analysis in this study. Note: Mean number of crypts scored are in brackets, images (A-C) shown are of the same magnification and the error bars are standard deviations. (TIF) [file pone.0078519.s007.tif]

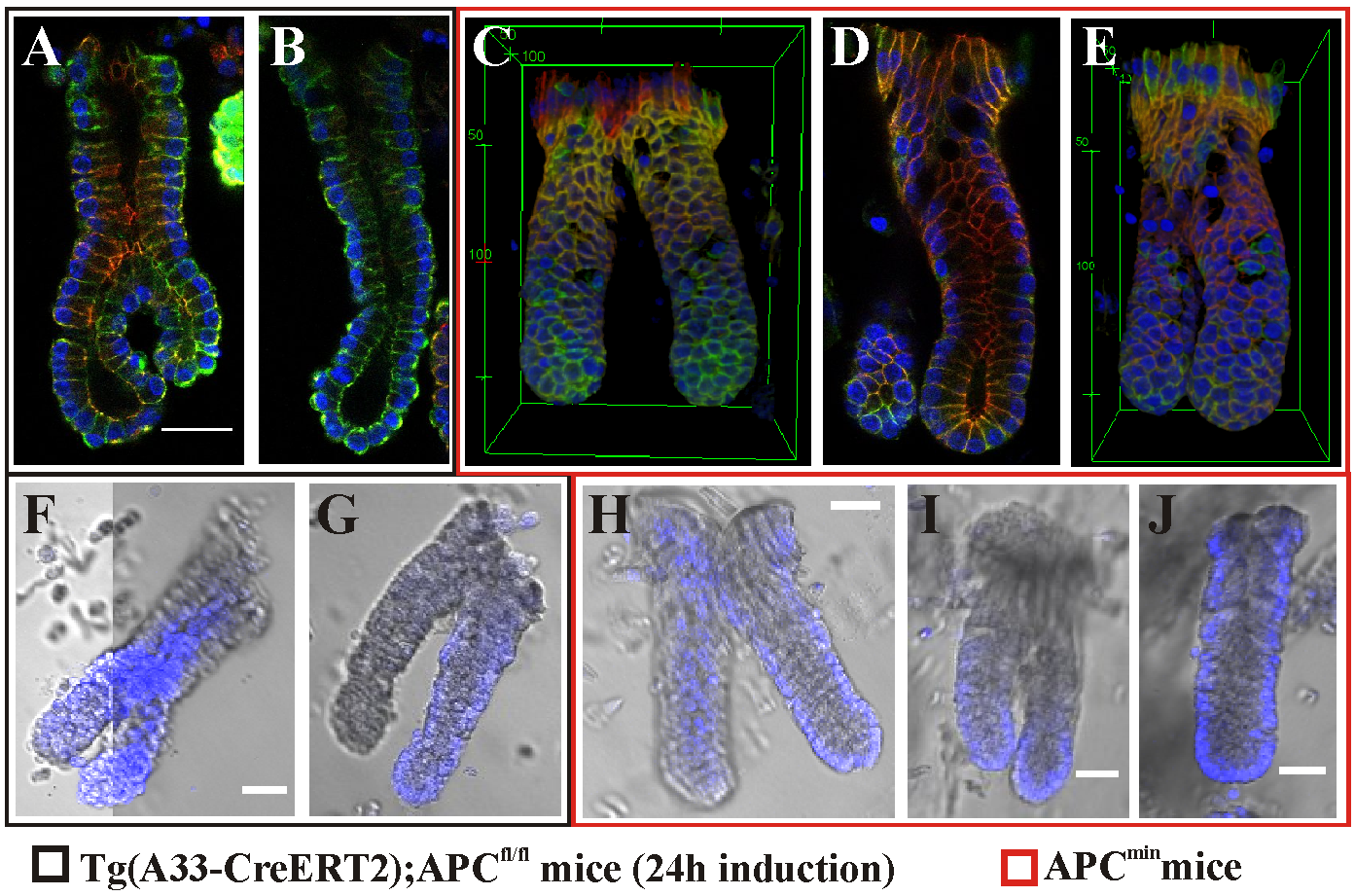

Supplement: Figure S6 — Morphology and β-catenin/E-cadherin distribution of isolated crypts from APCmin/+ and Tg(A33-CreERT2);Apcfl/fl mice. Confocal images of crypts isolated from Tg(A33-CreERT2);Apcfl/fl (A, B, F and G) and APCmin/+ (C-E, I-J; 24h induction with Tamoxifen to induce APC truncation) mice. No aberrant crypts with multiple crypt buds were identified, however, the heterogeneity of β-catenin and E-cadherin distribution was also observed in these crypts. Crypt shape appears similar to normal C57BL/6 mice with the exception of the Tg(A33-CreERT2);Apcfl/fl crypts which have an enlarged basal lumen (see A and B). Note: (A-E) Immunofluorescent confocal composite images for DAPI (blue), β-catenin (green) and E-cadherin (red), (F-G) confocal composite phase contrast image with DAPI. 3D volumetric reconstructions are shown in C and E. Scale bar: 30µm. (TIF) [file pone.0078519.s008.tif]
